# Estimating lighting direction in scenes with multiple objects

**DOI:** 10.3758/s13414-023-02718-0

**Published:** 2023-08-01

**Authors:** Lindsay M. Peterson, Daniel J. Kersten, Damien J. Mannion

**Affiliations:** 1 School of Psychology, UNSW Sydney, Sydney, NSW 2052 Australia; 2 Department of Psychology, University of Minnesota, Minneapolis, MN USA

**Keywords:** Illumination perception, Equivalent noise, Shape identification, Ensemble perception, Psychophysics

## Abstract

To recover the reflectance and shape of an object in a scene, the human visual system must account for the properties of the light illuminating the object. Here, we examine the extent to which multiple objects within a scene are utilised to estimate the direction of lighting in a scene. In Experiment 1, we presented participants with rendered scenes that contained 1, 9, or 25 unfamiliar blob-like objects and measured their capacity to discriminate whether a directional light source was left or right of the participants’ vantage point. Trends reported for ensemble perception suggest that the number of utilised objects—and, consequently, discrimination sensitivity—would increase with set size. However, we find little indication that increasing the number of objects in a scene increased discrimination sensitivity. In Experiment 2, an equivalent noise analysis was used to measure participants’ internal noise and the number of objects used to judge the average light source direction in a scene, finding that participants relied on 1 or 2 objects to make their judgement regardless of whether 9 or 25 objects were present. In Experiment 3, participants completed a shape identification task that required an implicit judgement of light source direction, rather than an explicit judgement as in Experiments 1 and 2. We find that sensitivity for identifying surface shape was comparable for scenes containing 1, 9, and 25 objects. Our results suggest that the visual system relied on a small number of objects to estimate the direction of lighting in our rendered scenes.

## Introduction

Recovering the intrinsic properties of an object in a scene, such as surface reflectance and shape, requires accounting for the prevailing lighting conditions. Although our perception of illumination has received insufficient psychophysical examination (Gilchrist, 2006; Schirillo, 2013), there is evidence that the visual system infers the lighting conditions in a scene. Observers can estimate the properties of a light source based on an object’s appearance (Kartashova, Sekulovski, de Ridder, te Pas, & Pont, 2016; Kartashova, de Ridder, te Pas, & Pont, 2018; Koenderink, Pont, van Doorn, Kappers, & Todd, 2007) and these estimates are evident in observers’ perception of surface reflectance (Boyaci, Maloney, & Hersh, 2003; Boyaci, Doerschner, & Maloney, 2004). Furthermore, the lighting conditions at different spatial locations in a scene can be accounted for when judging surface reflectance (Gilchrist, 1977, 1980; Mizokami, Ikeda, & Shinoda, 1998), with the observers relying on information given by multiple lighting cues (e.g., specular and non-specular objects) within a scene to make their judgements (Boyaci, Doerschner, & Maloney, 2006; Snyder, Doerschner, & Maloney, 2005).

**Fig. 1:**
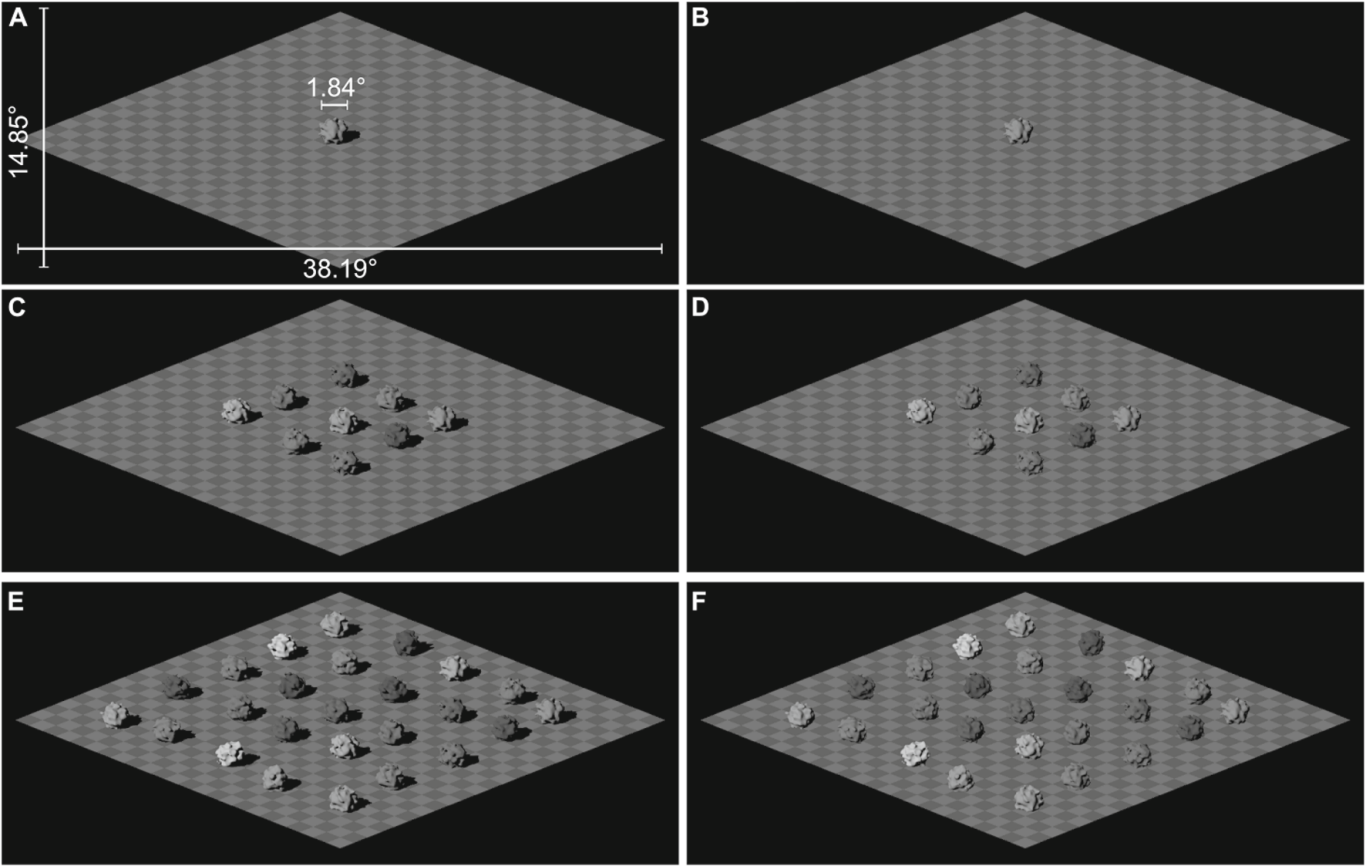
Examples of the stimuli from Experiment 1. Participants were presented with images of scenes containing 1, 9, or 25 objects and the objects were rendered either with (**A**, **C**, **E**) or without (**B**, **D**, **F**) cast shadows. The light source azimuth is $-45.25^\circ$ in each panel. The white labels in **A** show the dimensions of the stimulus in degrees of visual angle. See Fig. 6B for examples of an individual object illuminated from different azimuths

This apparent reliance on multiple objects indicates that some form of spatial integration may be occurring when observers are estimating the lighting conditions in a scene, similar to an ensemble or summary statistic (Haberman and Whitney, 2012; Pont, 2019; Sanders, Haberman, & Whitney, 2008; Whitney and Yamanashi Leib, 2018). Potential evidence for this suggestion comes from a conference abstract by Sanders et al. (2008), and described further by Haberman and Whitney (2012), who investigated whether the visual system has an ensemble representation of cast shadows (which is analogous to light source direction). Observers were presented with images of rendered geometric objects that were illuminated from a particular direction and were asked to judge the average orientation of the cast shadows for a set of objects. The accuracy with which observers completed the task implied that the estimated shadow orientation for individual objects within an image were integrated to estimate the mean shadow orientation. However, similar levels of accuracy were reported for the control images in which the cast shadows were perceived to be surface paint. As such, observers may have made their judgements without an explicit representation of light source direction; the average “shadow” orientation could have been computed without actually integrating multiple local estimates of shadow orientation. Therefore, the results from Sanders, Haberman, & Whitney (2008) are inconclusive in regards to the visual system utilising multiple objects within a scene to estimate lighting direction.

Here, we focus on examining the extent to which estimates of the direction of lighting in a scene are informed by multiple objects within that scene. If estimates of lighting direction are informed by multiple objects, we would expect to see a positive relationship between the number of objects in a scene and the number of objects that are relied upon to estimate lighting direction. This assumption is based on a trend reported for ensemble perception in which the number of integrated samples (i.e., the number of stimulus elements used to make a judgement about the stimulus) tends to increase as the number of stimulus elements increases (Whitney and Yamanashi Leib, 2018). If the visual system does combine multiple local samples to estimate lighting conditions in a scene, an observer should use more objects to judge light source direction in Fig. 1E compared to Fig. 1C and A, for example. As each local estimate will have some degree of noise associated with it, relying on multiple local estimates will result in greater precision compared to relying on a single local estimate. As a consequence, precision at estimating the direction of a light source should improve as more objects are added to a scene. Such an effect of set size has been found for ensemble perception; for example, Robitaille and Harris (2011) reported that larger set sizes were associated with improved accuracy when observers judged the mean size and orientation of a set of circles and tilted bars, respectively.

In three experiments, we measured participants’ ability to estimate the direction of lighting in scenes that contained a varying number of objects. In Experiment 1, participants were presented with scenes containing 1, 9, or 25 objects and indicated whether they perceived the scene as illuminated from the left or right, relative to their viewpoint. To further understand our results in Experiment 1, we used an equivalent noise paradigm in Experiment 2 to measure the number of objects used to estimate the average light source direction in a scene. To probe any potential differences between implicit and explicit judgements of lighting direction, participants completed a shape identification task in Experiment 3 that required an implicit judgement of lighting direction (rather than explicit, as in Experiments 1 and 2).

## Experiment 1

The aim of Experiment 1 was to examine the extent to which light source direction discrimination depends on the number of visible objects within a scene. Participants were presented with images of scenes that contained 1, 9, or 25 objects and asked to indicate whether the scenes were illuminated from the left or right, relative to their viewpoint. We also manipulated whether the scenes were rendered with or without cast shadows to examine the contribution of cast shadow information to any changes in discrimination sensitivity associated with variations in set size. If the visual system does use multiple samples to estimate the direction of lighting in a scene, performance on the light source direction discrimination task should benefit from an increasing number of objects in a scene.

### Methods

#### Participants

Thirty-two participants (18 female and 14 male; median age of 19 years) with self-reported normal or corrected-to-normal vision completed the experiment. The majority of participants (27/32) were 21-years-old or younger. Participants were recruited from a database of undergraduate students enrolled in a first-year psychology course at UNSW Sydney. Participants gave informed and written consent prior to beginning the experiment and experimental procedures were approved by the Human Research Ethics Advisory Panel at the School of Psychology, UNSW Sydney. All participants were naïve to the purpose of the experiment.

#### Apparatus

The experiment was run in three similar testing booths. In each booth, the experiment was presented on a Display++ LCD monitor (Cambridge Research Systems, Kent, UK). Each monitor had a spatial resolution of $1920 \times 1080$ pixels, temporal resolution of 120Hz, mean luminance of 60 cd/m$^2$, a linear relationship between graphics card signal and luminance, and a 10-bits per pixel luminance output resolution. Participants viewed the monitor, in an otherwise darkened booth, from a distance of approximately 60cm for a total visual angular subtense of approximately $66^\circ \times 37^\circ$. The stimuli for the experiment were created with POV-Ray (Version 3.7; https://www.povray.org/). The experiment was implemented using PsychoPy (Peirce, 2007, 2008), and analyses was performed using NumPy (Harris et al., 2020), SciPy (Virtanen et al., 2020), and PyMC (Salvatier, Wiecki, & Fonnesbeck, 2016).

#### Stimuli

The stimuli were rendered images of scenes containing 1, 9, or 25 objects (see Fig. 1 for examples). The geometry of the objects was created by applying a “bumpy” texture (f_bumps from POV-Ray’s library) to the surface of a sphere. This created blob-like objects with random surface curvature. The size of the objects was selected so that the objects did not occlude one another. The objects were situated on a flat checkerboard surface (with checks of 15% and 25% reflectance), illuminated by a single directional light source.

We rendered 100 instances of each combination of set size, cast shadows, and light source azimuth. The scene was captured with an orthographic camera that had an elevation of $23.2^\circ$ and was pointed towards centre of the scene. The scale and amplitude of the bump surface texture were randomised for each object in a scene. All of the objects had a diffuse (Lambertian) reflectance that was randomly chosen from a Beta distribution ($\alpha = 1.5$, $\beta = 6.5$), limited to the range 5% reflectance and 80% reflectance, to mimic the distribution of reflectances that is found in natural scenes (Attewell and Baddeley, 2007). The elevation of the light source was fixed at $40^\circ$ and the azimuth varied across renderings. For the no-cast-shadows condition, the light source was artificially prevented from casting shadows.

We chose to present scenes that contained 1, 9, and 25 objects as these set sizes allowed us to adjust the number of objects on the checkerboard surface while maintaining a regular spatial arrangement. The location of the objects in the scene was specified by a $5 \times 5$ grid. An object was placed in the centre of the grid for all set size conditions. Additional objects were added to the grid in the inner and outer ring surrounding the central object for the 9-object and 25-object condition, respectively. A small amount of jitter was added to the position of each object on the checkerboard surface, which meant that each object did not appear in the same exact location throughout the experiment.

#### Design and procedure

The experiment had a within-subjects design with factors of cast shadows (scenes rendered with or without cast shadows) and set size (1, 9, or 25 items). Participants completed the experiment in a single 45-minute session. Prior to beginning the experiment, participants were introduced to the task via a set of instructions that included a written explanation of the experiment and a short practice task. The practice task consisted of twelve trials (scenes with the light source azimuth as $-35.25^\circ$ and $+35.25^\circ$ for each condition) in which we expected participants to respond correctly unless they misunderstood the task. Participants were required to respond correctly on all of these trials before beginning the experiment.

The experiment consisted of 10 runs with 66 trials each, with a short rest break between each run. On each trial, the stimulus was presented for 600ms at full visibility, with 100ms ramp in and out from the black background, followed by the response prompt: “Was the scene lit from the left or right? Press the ‘left’ arrow key for left or the ‘right’ arrow key for right”. Participants received feedback on the correctness of their response in the form of a tick or cross appearing briefly on the screen before the subsequent trial began.

The experiment had 660 trials in total. Each condition had 100 trials that were randomly interleaved throughout the experiment. For each condition, there were additional 10 ‘catch’ trials in which the illumination angle was randomly chosen to be either $-70.25^\circ$ or $+70.25^\circ$. The angle of illumination on each trial was selected using a Psi-marginal adaptive staircase procedure (Kontsevich and Tyler, 1999; Prins, 2013), with separate staircases for each condition. Participants’ responses were modelled using a cumulative Normal function, which describes the probability of a participant judging a scene as lit from the right for a given illumination angle. Possible stimulus levels ranged from $-70.25^\circ$ to $+70.25^\circ$ in steps of $0.5^\circ$, with $0^\circ$ being the observer’s vantage point. The Psi procedure targeted the spread (inverse slope) of the psychometric function and marginalised over the function’s midpoint to optimise the estimation of the spread, which was central to assessing the effect of our manipulations on discrimination sensitivity.

#### Data analysis

Participants were excluded from the analysis based on their catch trial performance. Four participants were excluded from the analysis because their accuracy on the catch trials was less than $90\%$. The analysis described below was carried out with the remaining 28 participants.

**Fig. 2:**
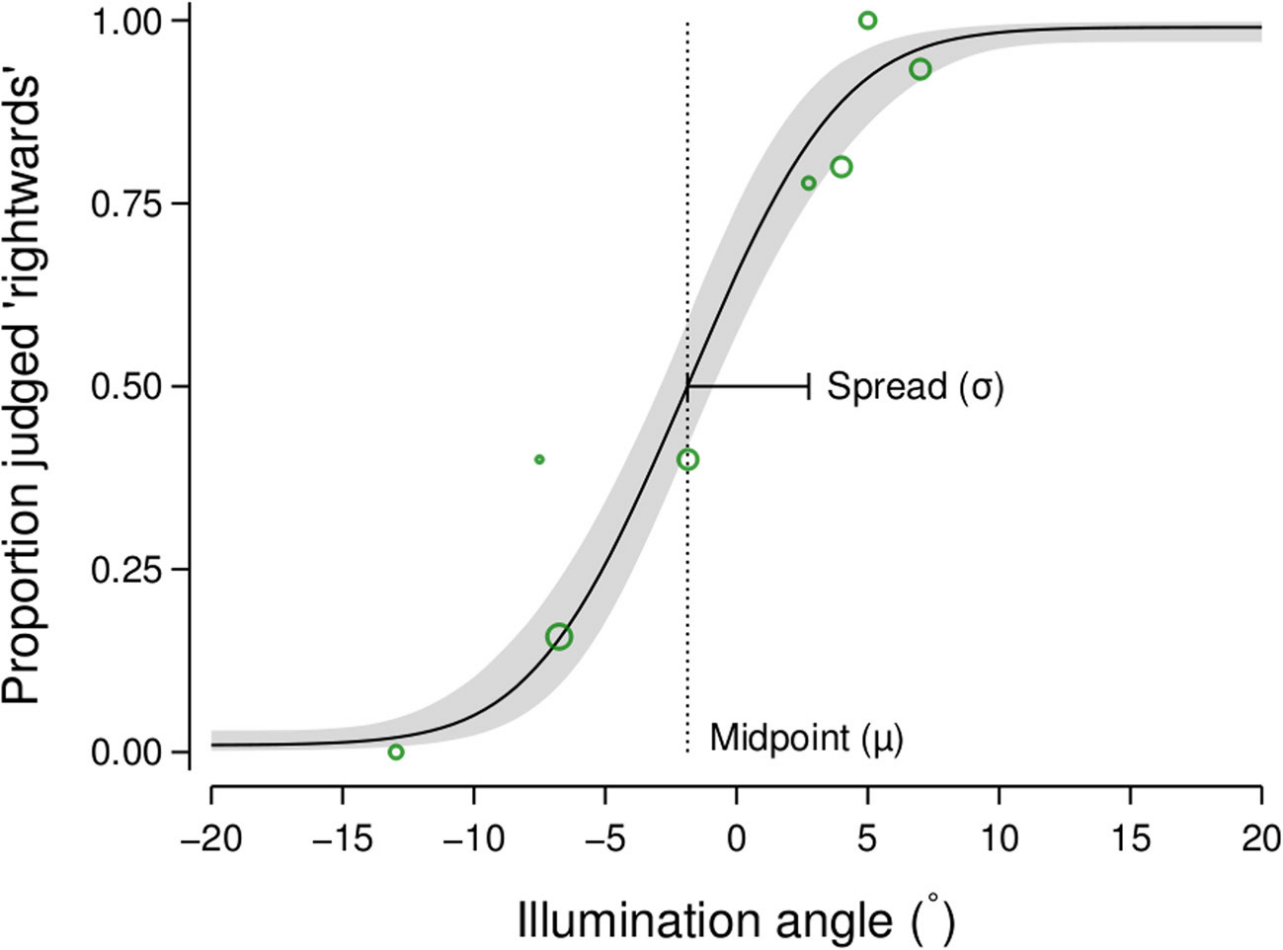
Example data and fitted psychometric function from a single condition (set size of 9 objects and with shadows present) for a representative participant in Experiment 1. The green circles show the mean proportion of rightwards judgements for trials where the illumination angle was within a given bin, with the size of the circles proportional to the number of trials in each bin. The solid line and grey region show the median and 95% credible interval of the psychometric function derived from the fitted parameters for this participant for this experimental condition

We used Bayesian statistical modelling to analyse the experimental data, with a focus on parameter estimation rather than hypothesis testing or model comparison (see Calin-Jageman and Cumming , 2019, for an overview of this approach) and our general approach to building the statistical models was motivated by Lee (2018); Wagenmakers et al. (2018), and Betancourt (2020). Participant performance in each experimental condition was modelled using a psychometric function, the parameters of which were estimated with a Bayesian generalised linear mixed model approach. The key parameter of interest was the spread of the psychometric function. The model for the psychometric function spread included fixed effects for the intercept, the main effect of cast shadows, the linear and quadratic main effects of set size rank, and the interaction between cast shadows and the set size rank effects. The quadratic main effect of set size rank was included in the model to allow for a non-monotonic effect of set size rank on the spread. We also included participant random effects in the model such that the fixed effects could vary across participants. The model for the midpoint of the psychometric function followed the same structure. A complete description of the statistical model can be found in Appendix A. Consistent with the Bayesian statistical modelling approach, we incorporated prior probabilities into the models which reflected what we expected to be the reasonable magnitude of the effect of our experimental manipulations (see Appendix A for an explanation of the specific priors placed on the model parameters). Figure 2 shows a summary of the observed data and the fitted psychometric function for a single experimental condition from a representative participant.

**Fig. 3:**
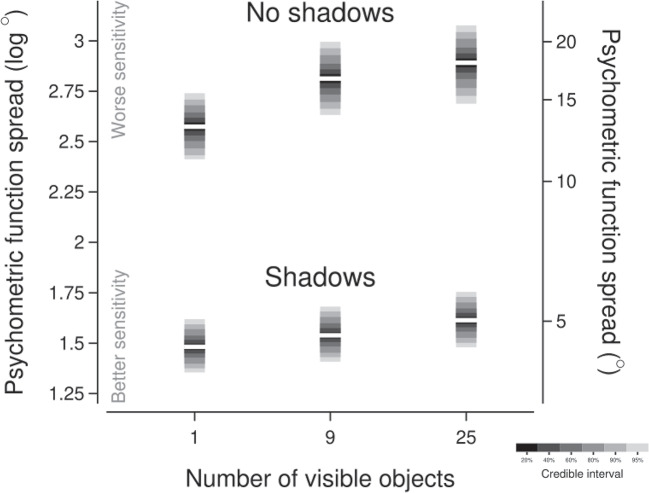
Summaries of the estimated posterior distributions for the spread parameter of the psychometric function for each experimental condition. The horizontal axis is the number of objects present in a scene (non-linearly spaced). The vertical axes show the spread of the psychometric function in log (left axis) and linear (right axis) units. Each posterior is shown as a ‘ribbon’ in which the central white bar represents the median of the posterior distribution and the surrounding bars represent credible intervals as shown in the legend

### Results

In this experiment, participants were shown an image of a scene and indicated whether they perceived the scene as lit from the left or right. We used a Bayesian linear mixed model approach to estimate the parameters of each observer’s psychometric function for each experimental condition. We compared the aggregated observed data and the posterior retrodictive samples from the fitted model and found that the model reproduced the patterns in the observed data with no major discrepancies (see Fig. 15 in Appendix A).

Our primary parameter of interest is the spread (inverse slope) of the psychometric function, where lower values are associated with steeper slopes and greater sensitivity and higher values are associated with shallower slopes and less sensitivity. Figure 3 summarises the estimated posterior distributions for the spread parameter for each of the six experimental conditions (whether cast shadows are present or absent for scenes with 1, 9, or 25 visible objects). If the presence of multiple objects increased the sensitivity with which an observer could discriminate the direction from which the scene was illuminated, we would expect the spread parameter to decrease with increasing numbers of visible objects. However, it is evident in Fig. 3 that the estimated spread actually increased, if anything, with increasing numbers of visible objects. The parameter capturing the linear component of the trend indicated that the spread increased by a factor of about 1.07 (posterior median; 95% credible interval: $\left[ 1.00, 1.14\right]$) with each increment in set size condition (i.e., from 1 to 9 and from 9 to 25 visible objects) when cast shadows were present, with a greater increase of a factor of about 1.17 (posterior median; 95% credible interval: $\left[ 1.09,1.25\right]$) when cast shadows were absent. The quadratic trend components were less influential, although Fig. 3 suggests that there may have been a saturation in the increase in spread with the set size condition when cast shadows were absent.

Presenting participants with scenes rendered without cast shadows worsened the sensitivity for illumination direction discrimination. The average spread across the set size conditions increased by a factor of about 3.36 (posterior median; 95% credible interval: $\left[ 2.86, 3.94\right]$) when cast shadows were absent compared to when they were present.

**Fig. 4:**
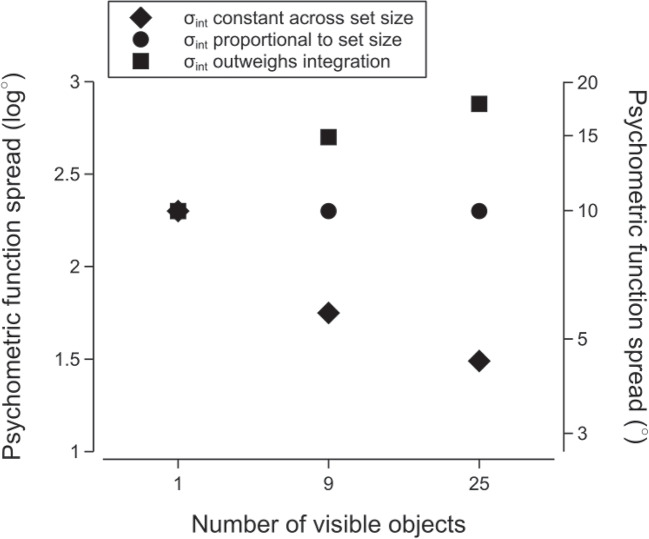
The possible effects of variations in internal noise on psychometric function spread, assuming integration of approximately the square root of the number of objects in a scene. If we assume that an observer has an internal noise of $10^\circ$ which remains constant across changes in set size, the spread should decrease with more objects in a scene. However, internal noise may vary with the number of objects in a scene. Internal noise may increase proportionally with set size and negate any benefit associated with integration, leading to no meaningful change in the spread across the set sizes (as depicted by the circle markers). Alternatively, internal noise could increase such that it overwhelms the benefits from integrating multiple objects, resulting in increases in the spread with more objects in a scene (as depicted by the square markers)

### Discussion

In this experiment, we aimed to measure light source direction discrimination for scenes that contained 1, 9, and 25 objects. We found that there was a small decrease in sensitivity as more objects were added to a scene and this decrease in sensitivity across set sizes was greater for scenes rendered without cast shadows. We also found that discrimination sensitivity decreased for scenes rendered without cast shadows, which is perhaps unsurprising given that previous research has identified cast shadows as an important cue for estimating light source properties (Boyaci et al., 2006; te Pas, Pont, Dalmaijer, & Hooge, 2017).

The potential decrease in discrimination sensitivity with set size could be due to a dependency of internal noise on set size—an effect that has been reported previously for ensemble coding. Dakin (2001) examined how varying the orientation of a group of textures affected judgements of the mean orientation of the textures, finding that increasing the number of texture elements in the stimulus led to an increase in internal noise. Dakin, Mareschal, and Bex (2005a) also reported an increase in internal noise with more stimulus elements when participants were asked to estimate the average motion direction of a group of dots.

It is possible that a similar relationship between set size and internal noise existed in the current experiment, confounding our interpretation of discrimination sensitivity as an indicator of participants relying on multiple objects to judge the direction of the light source. We have assumed that internal noise remains constant across set sizes and, therefore, precision should increase with more objects in a scene (as depicted by the diamond markers in Fig. 4). However, increases in internal noise with set size could outweigh any benefit associated with integrating multiple local estimates (as depicted by the circle and square markers in Fig. 4). In the current experiment, it is possible that participants were using multiple objects to judge light source direction but this was masked by increases in internal noise. That is, the decrease in discrimination sensitivity associated with increases in set size suggests that there may be a trade-off between precision and the reliance on multiple estimates, where any benefit associated with using multiple local estimates is outweighed by decreases in precision. The basis of the subsequent experiment is an equivalent noise paradigm, in which internal noise and the number of integrated objects can be estimated by presenting stimuli with varying levels of external noise (Barlow, 1956; Dakin, 2001; Dakin et al., 2005a; Pelli, 1990) .

**Fig. 5:**
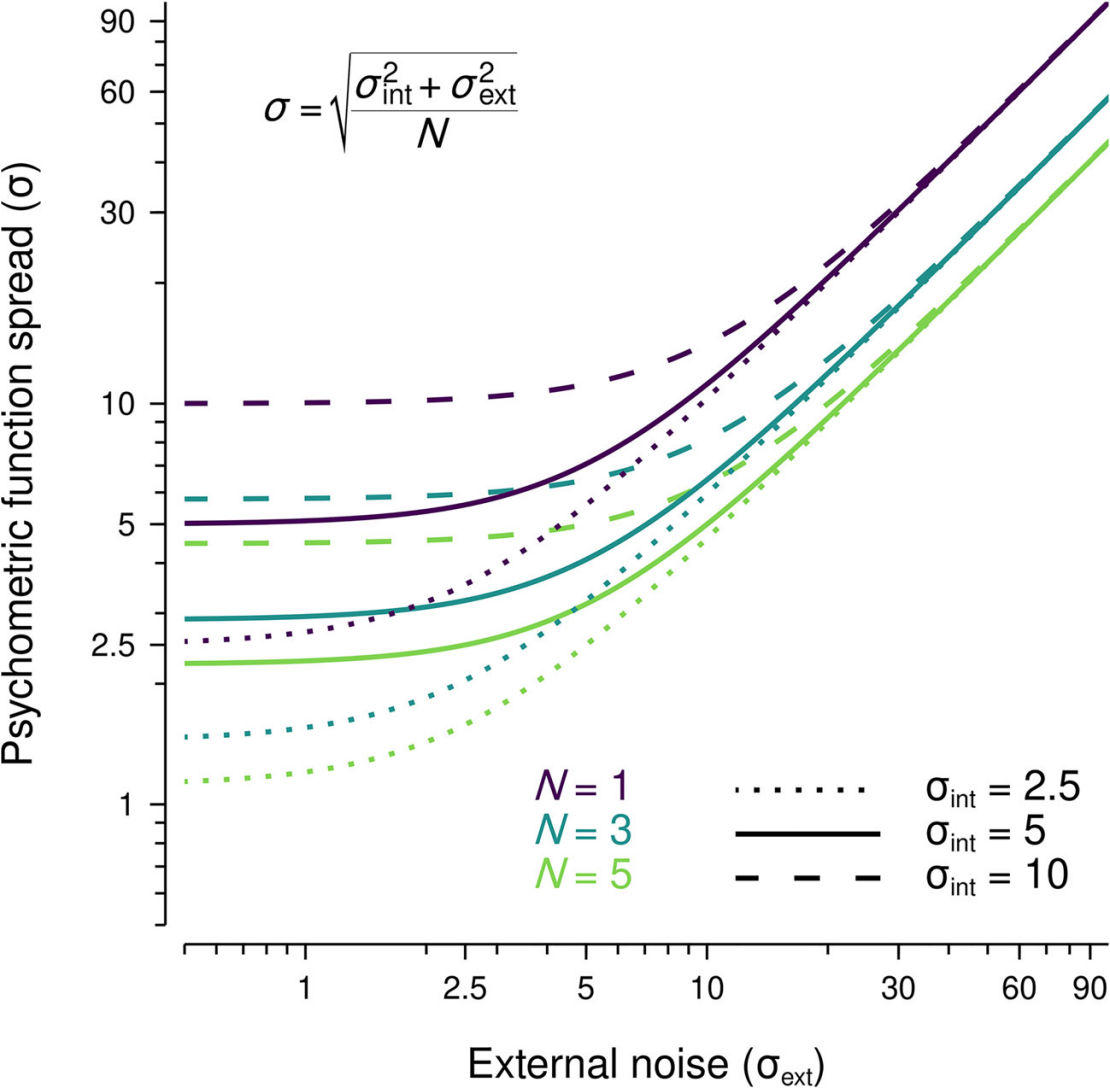
Examples of the relationship between external noise ($\sigma _{\text {ext}}$) and the spread of the psychometric function ($\sigma$) for different configurations of internal noise ($\sigma _\text {int}$) and the number of integrated samples (*N*). The line styles (dotted, solid, dashed) are associated with variations in internal noise and the line colours/brightnesses (purple/darker, blue/intermediate, green/lighter) are associated with variations in the number of integrated samples, with a given parameter configuration being the conjunction of these elements

## Experiment 2

In this experiment, we use an equivalent noise paradigm to measure the number of objects used to judge the average light source direction in a scene as well as participants’ internal noise. Within this paradigm, the spread of each participant’s psychometric function is defined as:

1 $$\begin{aligned} \sigma = \sqrt{\dfrac{\sigma _{\text {int}}^2 + \sigma _{\text {ext}}^2}{N}}. \end{aligned}$$

In Eq. 1, $\sigma _{\text {int}}$ is the internal noise, $\sigma _{\text {ext}}$ is the external noise, and *N* is the number of integrated objects. In an equivalent noise task, the presentation of stimuli with varying levels of external noise allows for estimation of internal noise and the number of integrated samples (Barlow, 1956; Dakin, 2001; Dakin et al., 2005a; Pelli, 1990) . Performance at low levels of external noise is determined by both internal noise and integration, and performance becomes increasingly determined by the number of integrated samples as external noise increases (Dakin, 2001), as depicted in Fig. 5.

**Fig. 6:**
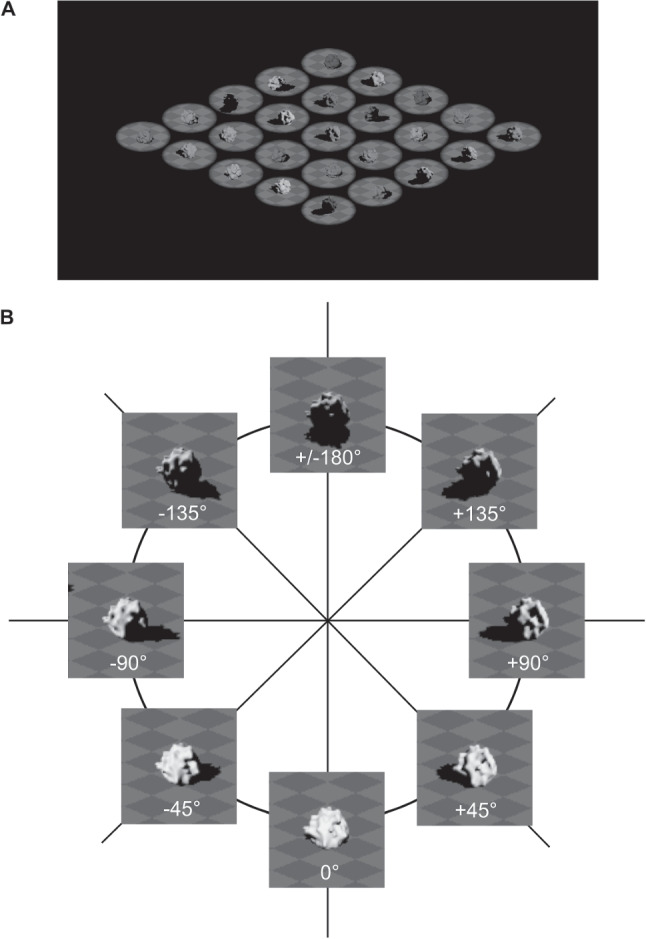
In Experiment 2, the light source azimuth was varied across objects to add external noise to the stimuli. An example of a scene with external noise is shown in **A**; azimuths for each object were drawn from a Normal distribution with a mean of $+50^\circ$ and standard deviation of $64^\circ$. In **B**, the object in each image has the same reflectance, surface curvature, and position in the scene. The white text label in each image indicates the azimuth of the light source illuminating the object. An azimuth of $0^\circ$ corresponds to the camera’s vantage point (i.e., frontal illumination) with negative and positive azimuths corresponding to leftwards and rightwards light source direction, respectively

Participants were presented with images of scenes that contained 9 and 25 objects and asked to the judge the average light source direction in a scene as leftwards or rightwards. We chose these set sizes to allow for comparisons to the results from Experiment 1, though the 1-object condition that was present in Experiment 1 was excluded from the current experiment due to constraints on the number of trials as well as its relative lack of informativeness within the equivalent noise paradigm. The light source azimuth for each object in a scene was drawn from a wrapped Normal distribution (Dakin, Mareschal, and Bex, 2005a) with a particular standard deviation—this allowed us to add external noise to the stimuli. A given object in a scene could be illuminated by a light source with an azimuth from the $-180^\circ$ to $+180^\circ$ range (examples of an object illuminated from different azimuths are shown in Fig. 6B). For example, in Fig. 6A, the light source azimuth for each object in the scene was drawn from a Normal distribution with a mean of $+50^\circ$ and a standard deviation of $64^\circ$.

### Methods

#### Participants

Thirty-eight participants (25 female and 13 male, median age of 19 years) with self-reported normal or corrected-to-normal vision participated in the experiment. The majority of participants (35/38) were 21-years-old or younger. The recruitment procedure was as described for the previous experiment.

#### Apparatus

The setup and implementation for this experiment was identical to what was reported for Experiment 1.

#### Stimuli

To allow for the addition of external noise to the stimuli, we first created a database of images of scenes with 25 objects, with each scene illuminated by a light source with an azimuth ranging from $-180^\circ$ to $+180^\circ$ in half-degree increments (total of 1,440 angles). The elevation of the light source was fixed at $40^\circ$ for all scenes. The geometry, reflectance, and placement of the objects in a scene were as described for Experiment 1. We rendered 100 instances of the images for each light source azimuth, varying object reflectance, geometry, and position jitter, which created a total of 144,000 images.

**Fig. 7:**
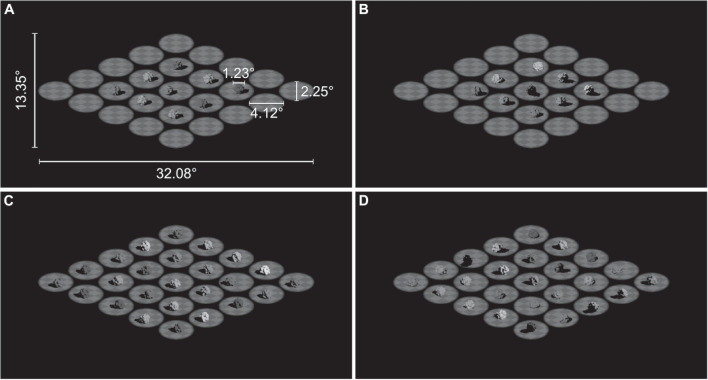
Examples of the stimuli from Experiment 2. The stimuli were images of scenes containing 9 or 25 objects. The white labels in **A** give the dimensions of the stimuli in degrees of visual angle. The average illumination angle is $-50^\circ$ in **A** and **B**, and $+50^\circ$ in **C** and **D**. External noise was added to the stimuli by varying the illumination angle for individual objects; **A** and **C** have $0^\circ$ of external noise, and **B** and **D** have $64^\circ$ of external noise

In the scenes shown to participants, the light source azimuth for each object was drawn from a Normal distribution where $\mu$ was the mean light source azimuth (hereafter referred to as the “mean illumination angle”) in the scene and $\sigma$ was the external noise level. To create these scenes, an image was selected from the database for each object based on an azimuth drawn from this distribution and a randomly chosen instance. A mask was applied to the selected image which removed all parts of the image apart from an oval-shaped aperture surrounding the object that was being added to the scene. This process was repeated for each object in a scene. For the 9-object condition (Figs. 7A and B), an “empty” image containing only the checkerboard surface, instead of an image from the database, was used for the outer object locations. We then combined the 25 masked images to create the scenes presented to participants; see Fig. 7 for examples.

#### Design and procedure

The experiment had a single factor design with set size (scenes containing 9 or 25 objects) manipulated within-subjects. Participants completed the experiment in a 45-minute session. The experiment began with a set of written instructions followed by a short practice task. The practice task consisted of twelve “easy” trials: mean illumination angle of $-90^\circ$ and $+90^\circ$, and external noise of $0^\circ$, $30^\circ$, and $48^\circ$ for each set size condition. Participants were required to make a correct response on all practice trials before beginning the experiment as an incorrect response would have indicated that the participant misunderstood the task.

The experiment had 10 runs with 66 trials per run and a 30-second rest break between each run. On each trial, the stimulus was presented for a total of 800ms; 600ms at full opacity plus a 100ms fade in and out from a black background. The stimulus presentation was followed by the response prompt: “On average, was the scene illuminated from the left or right? Press the ‘left’ arrow key for left or ‘right’ arrow key for right”. Participants received feedback on the correctness of their response in the form of a tick or cross appearing briefly on the screen before the next trial began.

The experiment had a total of 660 trials with 330 trials for each set size condition, including 30 ‘catch’ trials, which were randomly interleaved throughout the experiment. The mean illumination angle for the catch trials was either $-90^\circ$ or $+90^\circ$, with no external noise. The mean illumination angle and external noise level for each trial were chosen by a Psi-marginal adaptive staircase procedure (Kontsevich and Tyler, 1999; Prins, 2013), with separate staircases for each set size condition. Possible mean illumination angles ranged from $-90^\circ$ to $+90^\circ$ (with $0^\circ$ as the observer’s vantage point) and possible external noise levels were $0^\circ$, $30^\circ$, $48^\circ$, $64^\circ$, $80^\circ$, $96^\circ$, $112^\circ$, and $128^\circ$. The inclusion of very high levels of external noise allowed us to estimate the separate contributions of internal noise and the number of integrated objects to participants’ performance, as these parameters are confounded at low external noise levels (Dakin, Mareschal, & Bex, 2005b). We adjusted the Psi procedure to prevent the presentation of scenes with the same mean illumination direction for an extended number of trials. On a given trial, the mean illumination angle and external noise level were selected from within a range of stimulus values which were determined to be similarly informative of the psychometric function parameters. Furthermore, the sign of the selected angle was flipped if the five preceding trials all had mean illumination angles with the same sign.

Participant responses were modelled using a wrapped cumulative Normal function (Dakin, Mareschal, and Bex, 2005a) which describes the probability of judging the mean illumination direction in a scene as rightwards for a given mean illumination angle. The spread of the psychometric function was given by Eq. 1. As we were most interested in the $\sigma _{\text {int}}$ and *N* parameters, we used the Psi-marginal procedure to optimise the estimation of $\sigma _{\text {int}}$ and *N* and marginalise over the midpoint of the psychometric function.

#### Data analysis

Five participants were excluded from the analysis because their catch trial accuracy was below $90\%$. An additional two participants were excluded from the analysis due to unreasonably high estimates of internal noise ($>25^\circ$). The analysis described here was conducted with the remaining 31 participants.

**Fig. 8:**
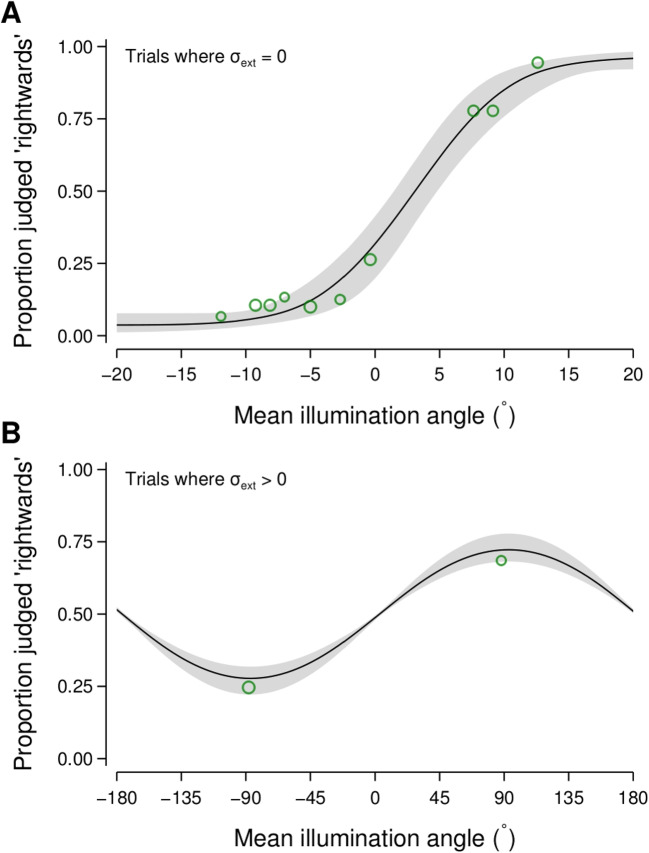
Example data and fitted psychometric function from a single condition (set size of 9 objects) for a representative participant in Experiment 2. The green circles show the mean proportion of rightwards judgements for trials where the mean illumination angle was within a given bin, with the size of the circles proportional to the number of trials in each bin, separately for trials without (**A**) and with (**B**) external noise. Note that the external noise levels for these trials were mostly $80^\circ$, $96^\circ$, and $112^\circ$. The solid lines and grey regions show the median and 95% credible intervals of the psychometric function derived from the fitted parameters for this participant for this experimental condition

We used a similar statistical approach to that taken for Experiment 1. The amount of internal noise, the number of integrated objects (parameterised as the exponent of the set size), and the midpoint of the psychometric function were each expressed using generalised linear mixed models (see Appendix B for a detailed description). Figure 8 shows a summary of the observed data and the fitted psychometric function for a single experimental condition from a representative participant.

### Results

In this experiment, participants were shown images of scenes in which individual objects could be illuminated from different directions. Participants were asked to judge whether the mean direction of illumination in each scene was leftwards or rightwards, relative to their viewpoint. We used an equivalent noise model to estimate the internal noise and the number of integrated objects that were consistent with the performance on the task. To check how well the model approximated the data, we compared the aggregated observed data and the posterior retrodictive samples and found that the model reproduced the patterns in the observed data with no major discrepancies (see Figs. 16 and 17 in Appendix B).

**Fig. 9:**
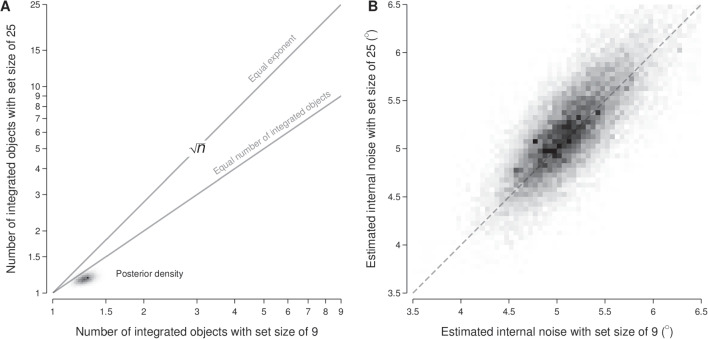
Joint posterior distributions for the two set size conditions (9 and 25 visible objects) for the number of integrated objects (**A**) and the amount of internal noise (**B**). In panel A, the labelled lines indicate the joint coordinates in which there is either an equal number of integrated objects for the two set sizes or the number of integrated objects is consistent with an equal exponent for the two set sizes (i.e., $9^x$ and $25^x$); note that both the horizontal and vertical axes are shown with log spacing

Figure 9A depicts the joint posterior for the estimated mean number of integrated objects for scenes with set sizes of 9 and 25 objects. As shown in the figure, the number of integrated objects was likely to be low: below about 1.5 when there were 9 visible objects and of comparable number (or lower, if anything) when there were 25 visible objects. Figure 9B depicts the joint posterior for the estimated mean amount of internal noise for scenes with set sizes of 9 and 25 objects; it shows that the mean amount of internal noise was around $5^\circ$ for both set sizes.

### Discussion

In the current experiment, participants were presented with images of scenes that contained 9 and 25 objects and indicated whether they perceived the average light source direction in the scene as leftwards or rightwards. Using an equivalent noise paradigm, we estimated each participant’s internal noise as well as the number of objects used to judge the average light source direction in a scene. We report that there was no systematic variation in internal noise or the number of objects used to judge average light source direction across the set size conditions.

As our analysis indicated that participants were using a small number of objects to judge average light source direction, we wanted to investigate which particular objects participants may have been relying on when making their judgements. To do this, we first computed the residuals as the difference between the observed judgements of whether the average illumination direction was rightwards and the probability of rightwards judgements according to the fitted model (Gelman, Goegebeur, Tuerlinckx, & Mechelen, 2000; Gelman, Hill, & Vehtari, 2020). If a particular object in the stimulus array was being used by participants as the basis of their judgements, we reasoned that this would manifest in a characteristic pattern to the residuals when examined as a function of the illumination angle of that particular object for trials in which the illumination angles across objects varied (that is, when the external noise was greater than zero). Specifically, the fitted model would under-predict the probability of a rightwards response when the illumination angle of the particular object is rightwards (and hence show positive residuals) and over-predict the probability of a rightwards response when the illumination angle of the particular object is leftwards (and hence show negative residuals). This pattern arises because the average illumination angle across objects, which is the basis for the fitted model, will not necessarily have the same sign as the illumination angle for a particular object on a given trial. For example, say if a particular object was being used by participants to make their lighting direction judgements. There will be some trials on which the object is lit from the left and the mean lighting across objects is from the right; the model would over-predict the propensity to respond ‘rightwards’ on those trials, due to its reliance on the mean lighting angle rather than the angle for this particular object. Conversely, there will be some trials on which the object is lit from the right and the mean lighting across objects is from the left; the model would under-predict the propensity to respond ‘rightwards’ on those trials. Hence, we can use this profile of under-prediction and over-prediction to identify the object(s) that are being utilised by participants in making their lighting direction judgements.

**Fig. 10:**
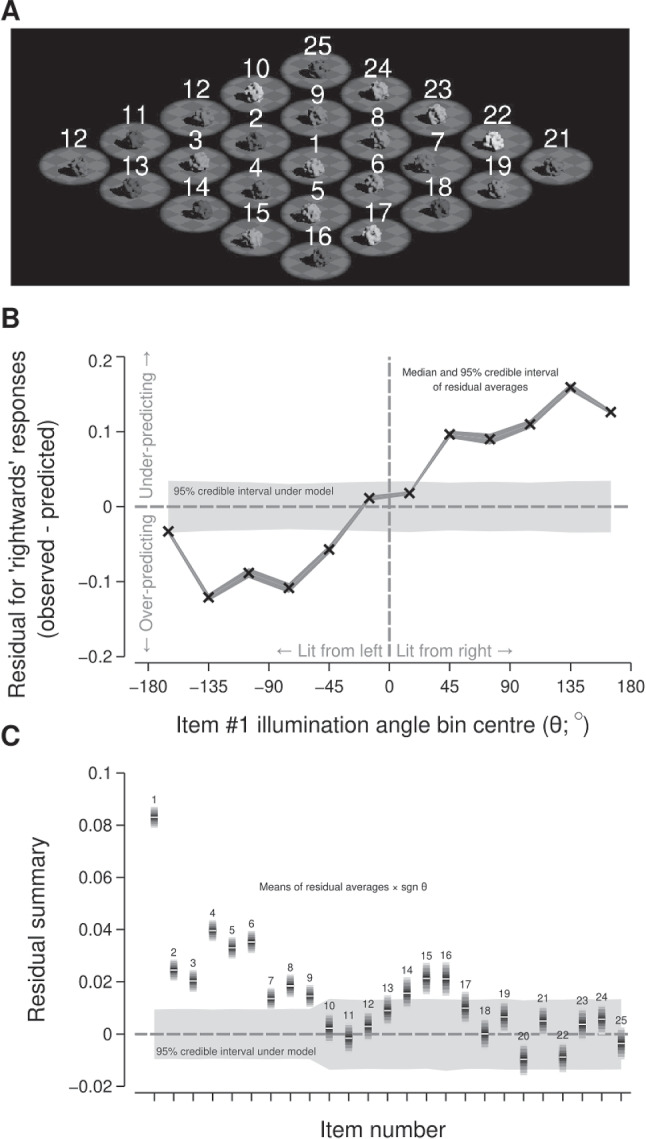
Analysis of the residuals as a function of the illumination angles for individual objects in the stimulus array. Panel **A** shows the assigned number for each object in the stimulus array. Panel **B** shows the average value of the residuals (vertical axis) for trials in which the illumination angle for object #1 was within a particular range (horizontal axis). The cross markers and dark grey ranges show the median and 95% credible intervals, respectively, for such averages. The light grey range shows the 95% credible interval for averages calculated using residuals computed with draws from the fitted posterior rather than from the observed responses. Panel **C** shows the summary value of the residuals (vertical axis) for each object (horizontal axis) as ribbon plots (see Fig. 3). The light grey range is as per panel **B**

We assigned each object in the array a number, as shown in Fig. 10A, and analysed the residuals as a function of each object’s trial-specific illumination angles. Figure 10B depicts the outcome of such an analysis for object #1, positioned in the centre of the object array, which shows an agreement with the pattern of residuals that would be expected if that particular object was being used to make the illumination direction judgements. To quantify this pattern, we reversed the sign of the residuals where the object illumination angle was negative and then averaged over the object illumination angles to give a summary of its adherence to the expected pattern of residuals. As shown in Fig. 10C, object #1 was clearly the object that had the highest summary score—suggesting that participants often relied on the centre object to estimate average light source direction. Florey, Clifford, Dakin, and Mareschal (2016) reported a similar result: when asked to judge the average gaze direction of a set of faces, observers tended to rely on the central items in the stimulus when making their judgements. A possible explanation for this reliance on the central items in the stimuli is that observers seem to focus on the centre of a computer screen when viewing stimuli (Tatler, 2007; Tseng, Carmi, Cameron, Munoz, & Itti, 2009).

In Fig. 10C, the grey band shows the 95% credible interval of the summary obtained by calculating the residuals using draws from the fitted model rather than the observed responses—which provides an indicative range of summary values that would be expected under the model. Comparing the summaries from the objects against such a band reveals two other sets of objects that have residuals that suggest that participants may have been using such objects when making their judgements. The first set consists of objects #4, #5, and #6, which lie immediately below the centre object in the object array. The second set, with a lower summary score, consists of objects #2, #3, #7, #8, and #9, which lie horizontally-adjacent to and above the central object, and objects #15 and #16, which lie in the lower outer ring of the object array. This suggests that participants may also have used objects surrounding the central object, particularly those positioned below the central object, when making their judgements. We speculate that these lower objects may have been utilised more frequently due to being inferred as being closer in depth to the observer and therefore more relevant to the egocentric illumination direction judgement.

A key component of this experiment was the presentation of stimuli with high ‘external’ noise, imposed by drawing the illumination angle of each object at random from a distribution with a large standard deviation (see Fig. 6). This approach was a methodological necessity that allowed for the number of integrated objects to be estimated separately from the level of internal noise (see Fig. 5). However, its interpretation assumes that the strategy adopted by observers is consistent across variation in external noise (Allard and Cavanagh, 2012)—which may be particularly questionable here given the ecologically-unusual scene that is produced with high levels of external noise. Although we consider the integration of such a wide range of lighting directions to be an unlikely requirement in everyday perception, we view this task as a situation in which suitably-instructed observers can voluntarily deploy an integration mechanism (if present) that operates in natural vision (under conditions in which there is less variation in lighting directions) within this artificial situation.

The current experiment aimed to measure participants’ internal noise and the number of objects used to judge the average light source direction in a scene. We report that participants used between 1 and 2 objects to judge average light source direction, regardless of whether the scene contained 9 or 25 objects. Internal noise was also similar across set sizes. Taken together with the results from Experiment 1, it seems that the visual system relies on a small number of local samples when estimating the direction of lighting in a scene.

## Experiment 3

In this experiment, we investigated whether differences in implicit and explicit judgements of light source direction could explain the lack of a set size effect reported in the previous two experiments. As noted by Murray and Adams (2019), implicitly estimating the properties of a light source is important for perceiving surface reflectance and shape but an explicit estimation may not be as important. Perhaps the visual system does utilise multiple local estimates of lighting direction when accounting for the effects of lighting direction on an object’s appearance but this was not evident in the previous two experiments as participants were making an explicit judgement, rather than an implicit one.

**Fig. 11:**
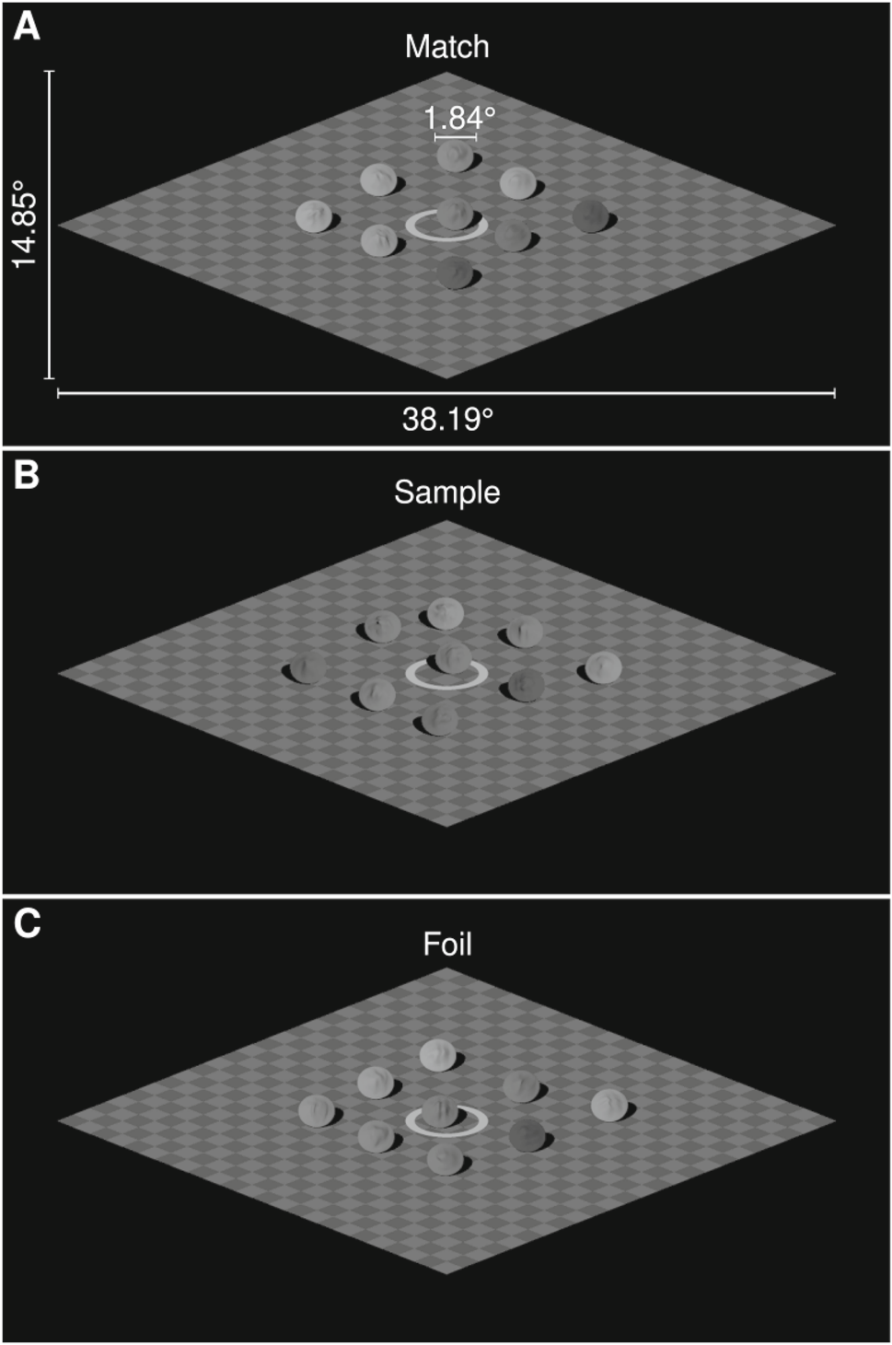
Examples of the stimuli from Experiment 3. The stimuli were rendered scenes containing 1, 9, or 25 objects. The white labels in panel **A** give the dimensions of the stimuli in degrees of visual angle. The stimuli shown here are examples of scenes that would be presented on a trial for the 9-object condition and incongruent illumination condition. Panel **A** is the match, panel **B** is the sample, and panel **C** is the foil. The stimulus generation process is described in the Stimuli section. The centre objects in each scene share the same orientation, reflectance, and ‘potato’ instance. The foil has a sinusoid distortion with an amplitude of approximately 0.117. The light source has an azimuth of $-30^\circ$ in **A** and **C** and an azimuth of $+30^\circ$ in **B**

In the current experiment, participants completed a match-to-sample task in which they were shown images of three scenes (a sample, a match, and a foil) that contained 1, 9, or 25 objects. Participants judged whether the shape of the centre object in the sample scene (e.g., Fig. 11B) was the same as the shape of the centre object in the match (e.g., Fig. 11A) or the foil scene (e.g., Fig. 11C). The scenes contained the same central object, however, the shape of the central object in the foil scene was distorted such that it was the odd one out. Participants’ accuracy (i.e., correctly selecting the match scene) should decrease as the amount of distortion applied to the foil object decreases and the foil object appears to be more similar in shape to the sample object. We also manipulated illumination incongruency such that the sample scene could be illuminated from the same or different direction as the match and foil scenes. If multiple objects are relied upon when implicitly estimating light source direction, precision at estimating light source direction should improve with more objects in a scene. Such an improvement in precision would be evident in participants’ responses, with shape identification based on shading cues improving with more objects in the scene.

### Methods

#### Participants

Thirty-eight participants (31 female and 7 male, median age of 19 years) with self-reported normal or corrected-to-normal vision completed the experiment. The majority of participants (34/38) were 21-years-old or younger. The recruitment procedures were as described for Experiment 1.

#### Apparatus

The experimental setup and implementation were as described for Experiment 1, with the addition of PyMesh (http://pymesh.readthedocs.io) which was used for creating the stimuli.

#### Stimuli

The geometry of the objects shown to participants was based on the stimuli used in Norman, Todd, and Orban (2004). First, we generated the mesh of a sphere (approximated by refining an icosahedron) and applied a vertical sinusoidal distortion with a randomly selected frequency from the range of 2 to 5 cycles per object and amplitude from the range 0.04 to 0.08 to the sphere’s vertices (where the amplitude values are a proportion of the radius of the sphere). The sphere was then rotated around the x-, y-, and z-axis and the amount of rotation on each axis was randomly chosen from the $0^\circ$ to $360^\circ$ range. This process of distorting and rotating the sphere was repeated a further four times, resulting in a potato-like object with bumpy surface curvature.

**Fig. 12:**
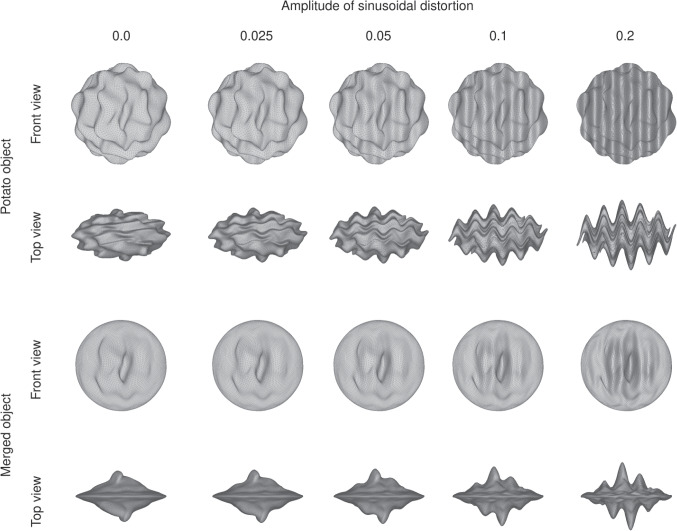
Examples of how varying the amplitude of the sinusoidal distortion affected the appearance of the stimuli in Experiment 3. The rows show the front and top views of a potato object before and after it was merged with a flattened sphere. Each column shows the potato object distorted with a sinusoid with an amplitude of 0.0, 0.025, 0.5, 0.1, and 0.2. The frequency of the sinusoid is 5.0 cycles per object

We created 100 instances of the potato object, randomly varying the frequency and amplitude of the sinusoidal distortions for each instance. Each potato’s vertices were then distorted with an additional vertical sinusoid with a frequency of 5 cycles per object and an amplitude ranging from 0.0 to 0.2 (amplitudes between 0.01 and 0.2 were logarithmically spaced, with 202 amplitudes in total). The potatoes were not rotated following this additional sinusoidal distortion. This resulted in a total of 20,200 potatoes; see Fig. 12 for examples of a potato object distorted with a sinusoid with different amplitudes. Each potato was then merged with the mesh of a flattened sphere. The weight of the potato in the merge varied from 1 in the centre of the flattened sphere to 0 towards the edges of the flattened sphere, with intermediate weights following a Gaussian profile. The merge created objects with varying internal surface curvature but with the same bounding contour (see Fig. 12 for examples).

We created scenes containing the merged objects using POV-Ray. The scenes contained 1, 9, or 25 objects which were placed on a flat checkerboard surface and tilted $50^\circ$ in depth away from the observer’s vantage point. A flat white ring that surrounded the centre object was also placed on the checkerboard surface. The reflectance and placement of the objects in a scene were as described for Experiment 1. The scenes were illuminated by a light source with an azimuth of $-30^\circ$ or $+30^\circ$, and an elevation of $40^\circ$. We rendered 100 instances of a scene for each combination of set size, light source azimuth, potato instance, the orientation of the centre object (whether the centre object was flipped upside down), and the amplitude of the sinusoidal distortion applied to the centre object. The position jitter of the objects was randomly varied for each instance of a scene. For instances of scenes containing 9 and 25 objects, the potato instance, reflectance, and orientation were varied randomly for each of the non-centre objects in a scene. Examples of the scenes are shown in Fig. 11.

**Fig. 13:**
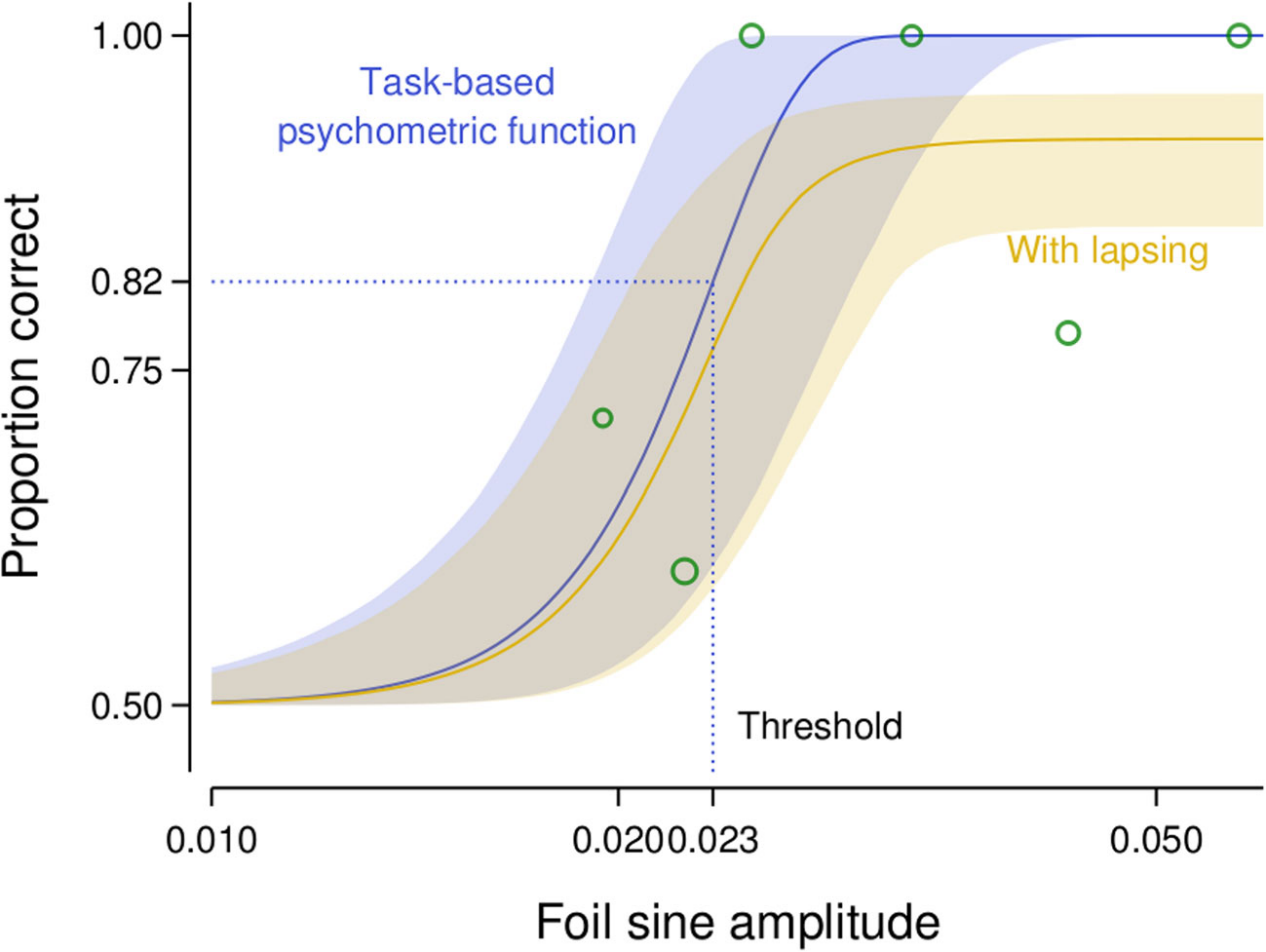
Example data and fitted psychometric function from a single condition (set size of 9 objects with congruent illumination) for a representative participant in Experiment 3. The green circles show the mean proportion of correct judgements for trials where the amplitude of the sinusoidal distortion applied to the foil object was within a given bin, with the size of the circles proportional to the number of trials in each bin. The solid lines and surrounding regions show the median and 95% credible intervals of the psychometric function derived from the fitted parameters for this participant for this experimental condition based only on task-related considerations (darker blue) and also when including lapsing (lighter yellow). The former is depicted because the key parameter of interest, the threshold, is defined as part of the task-based psychometric function. Note that the psychometric functions have wide credible intervals because the data collection procedure sacrificed precision of the slope to optimise estimation of the threshold

#### Design and procedure

The experiment had a within-subjects design with factors of set size (scenes with 1, 9, or 25 objects) and illumination incongruence (match and foil illuminated from the same or different direction to the sample). Participants completed the experiment in a single 45-minute session. Participants were introduced to the experiment via a set of written instructions and a short practice task. The practice task consisted of twelve “easy” trials (foil sinusoidal distortion amplitude of ${\sim }0.09$ and ${\sim }0.11$ for all conditions) in which we expected participants to respond correctly unless they misunderstood the task. Participants were required to respond correctly on all of the practice trials before beginning the experiment.

The experiment had 8 runs with 42 trials each, with a short rest break between each run. On each trial, the match, sample, and foil were each presented for 800ms (600ms at full visibility and 100ms ramp in and out from a black background) with a 800ms second gap between each interval. The centre object in each of the three intervals had the same potato instance, reflectance, and orientation, but the shape of the centre object in the foil interval was distorted with the sinusoidal amplitude for the trial. Depending on the illumination incongruence condition for the trial, the objects in the sample scene were illuminated from the same (congruent) or different (incongruent) direction to the objects in the match and foil scenes. The sample was always presented in the second interval; the order of presentation for the match and foil was randomly chosen on each trial. The presentation of the stimuli was followed by the response prompt: “Was the shape of the centre object in the second image the same as the shape of the centre object in the first or last image? Press the ‘left’ arrow key for the first image or ‘right’ arrow key for the last image”. Participants received feedback on their response in the form of a tick or cross appearing briefly on the screen before the next trial began.

The experiment had a total of 336 trials with 52 trials for each condition. There were an additional 4 ‘catch’ trials for each condition where the amplitude of the sinusoid distortion applied to the foil object was 0.2. For each trial, the amplitude of the sinusoid distortion was selected from the range 0.01 to 0.2 using a Psi-marginal adaptive staircase procedure (Kontsevich and Tyler, 1999; Prins, 2013), with separate staircases for each condition. Participant responses were modelled using a Weibull function (Kingdom and Prins, 2010) which describes the probability of a participant judging the shape of the match object to be the same as the shape of the sample object for a given sinusoidal distortion amplitude. The Psi procedure targeted the threshold of the psychometric function and marginalised over the slope of the function to optimise the estimation of the threshold.

**Fig. 14:**
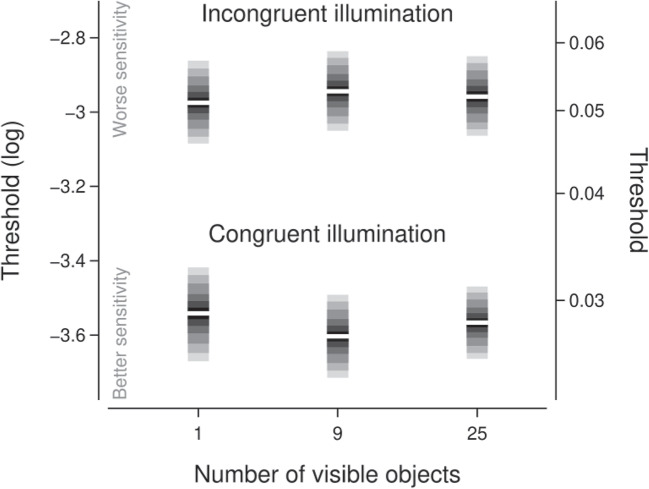
Effect of set size rank and illumination incongruence on identification thresholds. The horizontal axis is the number of objects present in a scene. The vertical axes show the threshold of the psychometric function in log (left axis) and linear (right axis) units. The display of the credible intervals is as per Fig. 3

#### Data analysis

Nine participants were excluded from the analysis as their catch trial accuracy was below $90\%$; the analysis described here was conducted with the remaining 29 participants. We used a similar statistical approach to that taken for Experiments 1 and 2, in which the threshold of the psychometric function was expressed using a generalised linear mixed model (see Appendix C for a detailed description). Figure 13 shows a summary of the observed data and the fitted psychometric function for a single experimental condition from a representative participant.

### Results

**Fig. 18:**
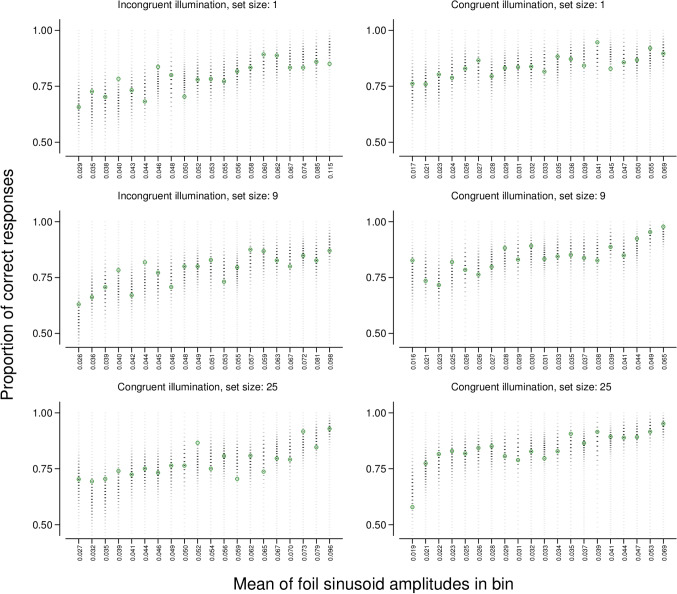
Summary of the observed data and the posterior retrodictive samples. Each panel corresponds to a particular set size and illumination incongruence condition. The horizontal axis represents the geometric mean of the foil sinusoid amplitudes in a bin, with 20 non-linear spaced bins in total. The amplitudes in each bin were chosen so that the summary in each bin was formed from an approximately equal number of trials. The vertical axis is the proportion of trials where participants responded correctly. The green circles represent the observed data. In each panel, the column of grey dashes at a given bin represents the number of trials with a foil sinusoid amplitude from that bin. The shade of grey of the dashes is inversely related to the number of posterior retrodictive samples at the associated response proportion, with darker shades corresponding to more samples and lighter shades corresponding to fewer samples. Note that the psychometric functions for each condition appear to be shallow as the Psi procedure marginalised over the slope of the function to optimise the estimation of the threshold

In this experiment, participants completed a match-to-sample task in which they were shown three scenes and asked to compare the shape of the centre object in each of the scenes. We used a Bayesian model to estimate the identification threshold for each experimental condition, with the threshold defined as the amplitude of the foil sinusoidal distortion which corresponded to around $82\%$ performance in correctly identifying the matching scene. Note that the amplitude values are a proportion of the radius of the originating sphere in the object creation process (see the Stimuli section for details). We compared the aggregated observed data and the posterior retrodictive samples to check how well our model approximated the data, finding that the model reproduced the patterns in the observed data with no major discrepancies (see Fig. 18 in Appendix C).

Figure 14 summarises the estimated posterior distributions for the threshold parameter for each of the six experimental conditions (whether the illumination was congruent or incongruent between the sample and the match/foil for scenes with 1, 9, or 25 objects). If the presence of multiple objects increased the capacity for an observer to discount the effects of illumination and recover the intrinsic shape of the target object, we would expected that the threshold would decrease with increasing numbers of visible objects. However, Figure 14 shows that there was very little change in thresholds with increasing numbers of visible objects. The parameter capturing the linear component of the trend indicated that the threshold changed by between a factor of about 0.94 and 1.06 (95% credible interval; median: 1.00) with each increment in set size condition (i.e., from 1 to 9 and from 9 to 25 visible objects), averaged over illumination congruence conditions.

Figure 14 also shows that presenting a sample scene with a different illumination direction to the match and foil scenes worsened identification performance. Across the set size conditions, thresholds in the incongruent illumination conditions increased by a factor of about 1.85 (posterior median; 95% credible interval: $\left[ 1.70, 2.00\right]$) relative to thresholds in the congruent illumination conditions.

### Discussion

The current experiment aimed to measure shape identification thresholds for scenes with 1, 9, and 25 objects, indirectly examining whether multiple objects are relied upon to estimate light source direction. We report that shape identification thresholds were similar across the set size conditions. Furthermore, our results suggest that the lack of a set size effect reported in Experiments 1 and 2 was not a consequence of participants making an explicit, rather than an implicit, judgement of light source direction.

The similarity in shape identification thresholds across the set sizes is consistent with Ho, Landy, and Maloney (2006), who reported that roughness constancy did not improve with more objects in a scene. In Ho et al. (2006), observers compared the roughness of two surfaces, with each of the surfaces embedded within its own scene. Consistent with the results reported here, adding cues to the spatial distribution of light in the scene (3D objects that varied in shape, colour, specularity, and position) did not improve discrimination performance. Ho et al. (2006) argued that the failure of these additional cues to improve roughness constancy was due to the observers’ reliance on cues that were not invariant to changes in illumination direction. Our results offer an alternative explanation: the additional cues did not lead to any improvement in roughness constancy because observers were potentially relying on only one object in the scene to estimate light source direction. Furthermore, the agreement between the results of the current experiment and those from Ho et al. (2006) suggests that the homogeneity of the objects in our scenes was not responsible for the lack of integration.

In addition to the set size manipulation, we also varied the congruence of the light source direction on each trial. We found that presenting scenes with incongruent illumination directions led to an increase in shape identification thresholds. This illumination incongruence effect is consistent with previous research which has reported that perception of surface shape is not constant across variations in light source direction (Caniard and Fleming, 2007; Christou and Koenderink, 1997; Khang, Koenderink, & Kappers, 2007; Tarr, Kersten, & Bulthoff, 1998).

In the present experiment, we continued to investigate whether the visual system uses multiple local estimates to judge light source direction. Using a match-to-sample task, we aimed to measure shape identification thresholds for scenes that contained 1, 9, and 25 objects. We reported that identification thresholds were similar across the set size conditions which suggests that participants were not utilising multiple objects to judge the direction of the light source illuminating a scene. This consistent with the results from the previous two experiments, indicating that the lack of a set size effect in those experiments was not a consequence of participants making an explicit (rather than implicit) judgement of light source direction.

## General discussion

The results of the three experiments presented here point towards the visual system relying on a limited number of objects in a scene to estimate the direction of lighting. In Experiment 1, we report that there was, if anything, a small decrease in participants’ sensitivity at discriminating light source direction as the number of objects in a scene increased. The results from Experiment 2 indicated that the number of objects used to judge average light source direction was similar for scenes that contained 9 and 25 objects. The apparent lack of an improvement in performance with set size in Experiments 1 and 2 does not seem to be due to any potential differences in implicit and explicit judgements of light source direction, based on the results of Experiment 3.

Our results contradict the suggestion that lighting direction may be represented as an ensemble or summary statistic (Haberman and Whitney, 2012; Pont, 2019; Sanders, Haberman, & Whitney, 2008; Whitney and Yamanashi Leib, 2018). A trend that has emerged in ensemble perception is that observers tend to integrate the square root of the total number of items in a stimulus when generating a summary statistic (Whitney and Yamanashi Leib, 2018). If lighting direction was represented in a similar manner to other ensembles, we would have expected to see a similar trend in the present study. Instead, we find that estimates of lighting direction are informed by 1 or 2 objects within a scene. As such, while there is some evidence of observers relying on multiple objects to estimate lighting direction, it seems that any spatial integration occurring is minimal and not consistent with ensemble representations of other visual stimuli.

The reliance on a small number of objects to estimate light source direction provides a potential explanation for the visual system’s apparent insensitivity to illumination inconsistencies in a scene. Ostrovsky, Cavanagh, & Sinha (2005) reported that observers struggle to locate an oddly illuminated target object in real and rendered scenes. Subsequent studies examining the visual system’s insensitivity to illumination inconsistencies within a scene reported similar results (Farid and Bravo, 2010; Ferwerda, Selan, & Pellacini, 2010; Lopez-Moreno, Sundstedt, Sangorrin, & Gutierrez, 2010; Nightingale, Wade, Farid, & Watson, 2019; Tan, Lalonde, Sharan, Rushmeier, & O’Sullivan, 2015). An oddly illuminated object in a scene would likely be missed if the consistency of local estimates of light source properties is not verified by the visual system (Ostrovsky et al., 2005). Such a lack of verification is consistent with Koenderink et al. (2007) who noted that when observers were adjusting the appearance of a probe object to “fit” into a scene, the lighting conditions which did not immediately surround the probe seemed to be ignored. The results from Experiment 2 indicate that 1 or 2 objects are relied upon to estimate lighting direction in a scene; this can be interpreted as 1 object being relied on for some scenes, while 2 objects informed estimates for other scenes. The former case supports Ostrovsky, Cavanagh, & Sinha ’s (2005) claim that a lack of verification of local estimates of lighting direction explains observers’ insensitivity to illumination inconsistencies in a scene. In the latter case, it is reasonable that an oddly illuminated object may still be missed if only two objects are integrated, particularly if estimates are spatially restricted (as suggested by the item analysis for Experiment 2).

It could be argued that the lack of an effect of set size on light source direction discrimination was related to our stimuli. The stimuli presented to participants in our experiments were images of simple scenes rendered without mutual illumination: we chose to keep the scenes simple as this was (to our knowledge) the first set of experiments to directly measure the number of objects used to estimate the direction of lighting in a scene. Perhaps the absence of mutual illumination, as well as the general simplicity of our scenes, could have made our scenes seem unrealistic and affected our results in some way. However, both Gilchrist (2018) and Motoyoshi and Matoba (2012) presented observers with realistic scenes yet found no evidence of the perceived reflectance of a target surface being influenced by the lighting conditions in the surrounding context. This is consistent with the results reported here; we would not expect the lightness of the target to be influenced by the surrounding context if there is very minimal (or no) spatial integration occurring when observers are estimating the lighting conditions in a scene. Even so, it is important to note that the generalisability of the results presented here is somewhat limited due to the simplicity of the scenes used in the three experiments. A potential direction for future research is to examine the how estimation of the lighting conditions in a scene may depend on the type of scenes presented to participants.

Additionally, the positioning of objects in our scenes may have affected the capacity for spatial integration. The variation in set size was achieved by surrounding the central objects with additional objects, and the number of objects within a scene was unpredictable across trials. This may have motivated observers to limit their spatial attention to the regions most likely to contain an object; an integration strategy that relies on local attention can reduce the accuracy with which summary statistics are computed (Chong and Treisman, 2005). Such a strategy may be consistent with the apparent over-reliance on the central object reported here for Experiment 2 (see Fig. 10). Alternatively, it could also be argued that the potential presence of peripheral objects could instead encourage observers towards a global attentional strategy. Given this potential influence of attention on spatial integration, further experimentation is necessary to determine the role of attention in estimating scene lighting.

There is a possibility that the lack of a set size effect was due to the short stimulus presentation time. We selected the presentation time based on practical limitations (e.g., time constraints) as well as ancedotal feedback from pilot participants, which suggested that there was sufficient time to view the scene on each trial. However, a potential explanation for the lack of a set size effect is that the short presentation time prevented participants from integrating as there actually was not enough time to analyse the whole scene. Ho et al. (2006) made a similar argument when discussing why the addition of multiple lighting cues to a scene did not improve roughness constancy, though further experimentation revealed that the initial result remained with a longer stimulus duration. Furthermore, evidence of spatial integration has been reported for a range of visual stimuli with similar presentation times (Whitney and Yamanashi Leib, 2018). As such, we consider the stimulus presentation time to be a possible but unlikely explanation for the minimal spatial integration reported here.

The overall aim of the experiments presented here was to investigate the extent to which estimates of direction of lighting in a scene are affected by the number of visible objects in the scene. The key conclusion of this study is that estimates of lighting direction are spatially restricted, with observers utilising a small number of objects in a scene to make their estimates. The current study casts doubt on the claim that lighting direction is represented as an ensemble or summary statistic; the results suggest that any integration is minimal and inconsistent with summary statistical representations of other visual stimuli.

## Figures and Tables

**Fig. 15:**
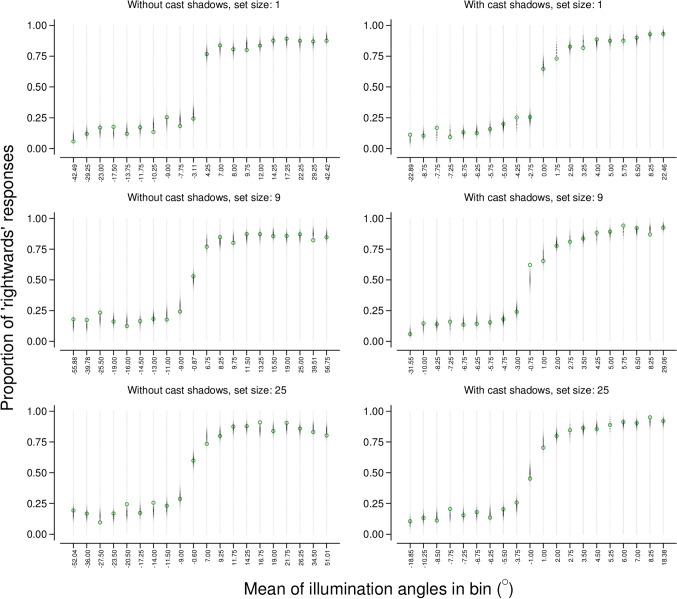
Summary of the observed data and the posterior retrodictive samples for each condition. Each panel corresponds to a particular cast shadow and set size condition. The horizontal axis represents the mean of the illumination angles in a bin, with 20 non-linear spaced bins in total. The angles in each bin were chosen so that the summary in each bin was formed from an approximately equal number of trials. The vertical axis is the proportion of trials where scenes were judged as illuminated from the right. The green circles represent the observed data. In each panel, the column of grey dashes at a given bin represents the number of trials with an illumination angle from that bin. The shade of grey of the dashes is inversely related to the number of posterior retrodictive samples at the associated response proportion, with darker shades corresponding to more samples and lighter shades corresponding to fewer samples

**Fig. 16:**
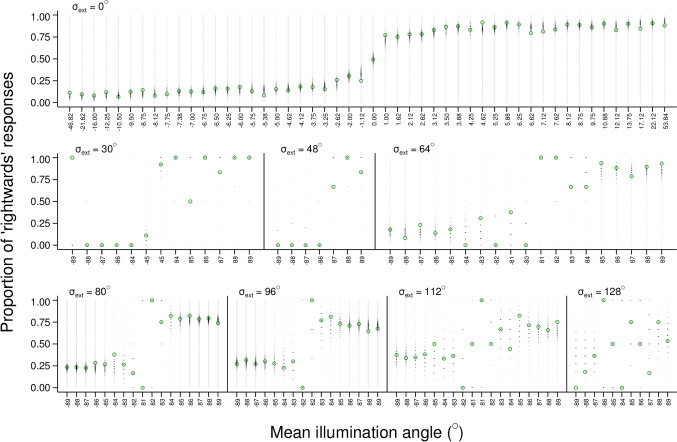
Summary of the observed data and the posterior retrodictive samples for the 9-object condition. Each panel corresponds to a particular external noise level. The horizontal axis is the mean illumination angle. Each horizontal axis tick in the $\sigma _{\text {ext}} = 0^\circ$ panel represents the mean of a group of mean illumination angles that were chosen so that the summary at each tick was formed from an approximately equal number of trials. Note that the horizontal axis ticks are non-linearly spaced. The vertical axis is the proportion of trials where the mean illumination direction was judged as rightwards. The green circles represent the observed data. The columns of grey dashes in each panel represent the number of trials at a particular mean illumination angle. The shade of grey of the dashes is inversely related to the number of posterior retrodictive samples at the associated response proportion, with darker shades corresponding to more samples and light shades corresponding to fewer samples

**Fig. 17:**
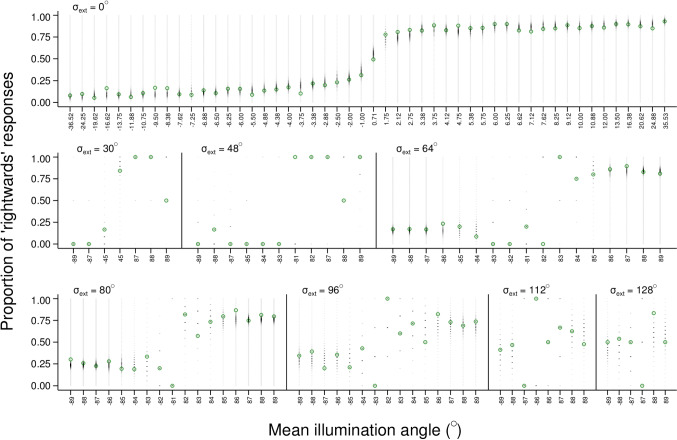
Summary of the observed data and the posterior retrodictive samples for the 25-object condition. See the caption of Fig. 16 for a description of the figure

## Appendix A: Experiment 1

### Statistical model

We assume that participant responses on the main task trials (*y*), whether the scene was judged as being illuminated from the right (1) or left (0), can be considered as draws from a Bernoulli distribution:

2 $$\begin{aligned} y_{ijkr} \sim \text {Bernoulli}\left( p_{ijkr}\right) \end{aligned}$$

where *i* indicates participants, *j* indicates cast shadow conditions, *k* indicates set size conditions, and *r* indicates repeats. We assume that the probability of a rightwards response (*p*) reflects a mixture of two processes: a process based on the trial stimulus and task and a process that is independent of the trial stimulus and task. We refer to the latter process as a ‘lapse’, and we assume that each participant has a propensity to lapse that we refer to as their lapse rate ($\lambda$). The probability of a rightwards response is given by a weighted combination of these two processes:

3 $$\begin{aligned} p_{ijkr} = \left( 1 - \lambda _i\right) p^t_{ijkr} + \lambda _i p^c. \end{aligned}$$

Here, $p^t$ is the probability of a rightwards response based on task-based factors and $p^c$ is the probability of a rightwards response under a lapse. We assume that participants are equally likely to respond with either response option during a lapse and hence fix $p^c$ to 0.5.

**Table 1:** Predictor values included in the model for the spread (Eq. 7) and the midpoint of the psychometric function

	No shadows	No shadows	No shadows	Shadows	Shadows	Shadows
	Set size: 1	Set size: 9	Set size: 25	Set size: 1	Set size: 9	Set size: 25
$x_1$	+0.5	+0.5	+0.5	-0.5	-0.5	-0.5
$x_2$	-0.5	0.0	+0.5	-0.5	0.0	+0.5
$x_3$	+0.5	-1.0	+0.5	+0.5	-1.0	+0.5
$x_4$	-0.25	0.0	+0.25	+0.25	0.0	-0.25
$x_5$	+0.25	-0.5	+0.25	-0.25	+0.5	-0.25

The lapse rate for each participant was informed by their responses to the catch trials, as any incorrect response on a catch trial was considered to be indicative of a lapse. We thus modelled the number of incorrect responses on the catch trials ($y^c$) as:

4 $$\begin{aligned} y^c_i \sim \text {Binomial}\left( 60, \lambda _i p^c \right) \end{aligned}$$

where *i* indexes the participant, 60 is the number of catch trials per participant, $\lambda$ is the lapse rate, and $p^c$ is the probability of an incorrect response under a lapse (assumed to be 0.5). We modelled the lapse rates using a generalised linear model with a logit link function:

5 $$\begin{aligned} \text {logit}\; \lambda _i = \beta _0^\lambda + \zeta _{0;i}^\lambda \end{aligned}$$

where $\beta _0^\lambda$ is the average lapse rate across participants and $\zeta _0^\lambda$ is a random effect that allows the lapse rates to vary across participants (both on the logit scale).

We modelled the probability of a rightwards response based on task-relevant factors ($p^t$) via a cumulative Normal psychometric function ($\Phi$):

6 $$\begin{aligned} p_{ijkr} = \Phi \left( \theta _{ijkr}, \mu _{ijk}, \sigma _{ijk} \right) . \end{aligned}$$

Here, $\theta$ is the illumination angle in the stimulus of a given trial, $\mu$ is the midpoint of the function, and $\sigma$ is what we refer to as the ‘spread’ of the function (with lower values relating to steeper slopes and higher values relating to shallower slopes).

We are primarily interested in the spread parameter ($\sigma$), which we modelled via a generalised linear model with a log link function:

7 $$\begin{aligned} \log \sigma _{ijk}= & {} \left( \beta _0^\sigma + \zeta _{0;i}^\sigma \right) x_0 + \left( \beta _1^\sigma + \zeta _{1;i}^\sigma \right) x_{1;j} \nonumber \\ {}{} & {} + \left( \beta _2^\sigma + \zeta _{2;i}^\sigma \right) x_{2;k} +\left( \beta _3^\sigma + \zeta _{3;i}^\sigma \right) x_{3;k}\nonumber \\{} & {} + \left( \beta _4^\sigma + \zeta _{4;i}^\sigma \right) x_{4;jk} + \left( \beta _5^\sigma + \zeta _{5;i}^\sigma \right) x_{5;jk}. \end{aligned}$$

Here, the *x* terms represent the predictors for the intercept ($x_0 = 1$), the main effect of cast shadows ($x_1$), the linear ($x_2$) and quadratic ($x_3$) effects of set size rank, and the interaction between cast shadows and the linear ($x_4$) and quadratic ($x_5$) effects of set size rank (see Table 1 for the specific predictor values). The $\beta ^\sigma$ coefficients represent the fixed effect of each predictor and the $\zeta ^\sigma$ coefficients are random effects that permits variation across participants. Note that we specified $\sigma$ in logarithmic units to prevent any negative spreads as we expected the probability of judging a scene as illuminated from the right to increase as the angle of the light source transitioned from negative to positive values.

A consequence of specifying $\sigma$ in logarithmic units is that the additive effects in Eq. 7 are multiplicative on a linear scale.

The midpoint of the psychometric function ($\mu$) represents the illumination angle at which an observer is equally likely to judge the scene as illuminated from the left or right. For an unbiased observer, the midpoint would be $0^\circ$. To allow for bias in observers’ responses, the midpoint was modelled analogously to the spread parameter ($\sigma$), except for the use of a linear rather than log link function.

### Statistical approach

We used a Bayesian framework (Lee, 2018; Wagenmakers et al., 2018; van de Schoot et al., 2021) to determine the posterior probability distribution of the statistical model parameters given our observed data (judgements of illumination direction on catch and non-catch trials). Reporting summaries of the posterior distributions is our primary method of communicating the outcomes of the study (Kruschke and Liddell, 2018), as our primary goal is to estimate the values of the parameters rather than to perform formal hypothesis testing or model comparison (Calin-Jageman and Cumming, 2019). We use the median as the measure of centrality and credible intervals (equal-tailed intervals, calculated using quantiles; Makowski, Ben-Shachar, & Ludecke 2019) as the measure of uncertainty when summarising the posterior distributions.

The Bayesian framework requires the specification of prior distributions for the model parameters. We adopted the strategy of providing weakly informative priors, where the informative aspect is typically on the approximate scale of the parameter values. The prior for the spread intercept ($\beta _0^\sigma$) was a Normal distribution with a mean of $\log \left( 10\right)$ and standard deviation of $\log \left( 2\right) /2$, as we expected discrimination sensitivity to be such that the spread of participants’ psychometric functions were around $10^\circ$ across all conditions. The prior for the main effect of cast shadows ($\beta _1^\sigma$) on the spread was a skewed-Normal distribution with a location of 0, scale of $\log \left( 4\right) /2$, and skew of 4, as we expected that rendering scenes without cast shadows would lead to increased psychometric function spreads compared to scenes rendered with cast shadows. We were particularly interested in $\beta _2^\sigma$ and $\beta _3^\sigma$ as these parameters represented the linear and quadratic effects of the set size manipulation on the spread of the psychometric function. The priors for $\beta _2^\sigma$ and $\beta _3^\sigma$ were each a Normal distribution centered around zero with a standard deviation of $\log \left( 2\right) /2$. The priors for the interaction effects ($\beta _4^\sigma$ and $\beta _5^\sigma$) on the spread were each a Normal distribution centered around zero with a standard deviation of $\log \left( 1.25\right) /2$. The priors for each of the parameters for the midpoint $\beta ^\mu$ were given by a Normal distribution with a mean of 0 and a standard deviation of 1, with the exception of the midpoint intercept parameter ($\beta _0^\mu$) which had a standard deviation of 2.5. The prior for the average lapse rate parameter ($\beta _0^\lambda$), on a logit scale, was given by a Normal distribution with a mean of $\text {logit}\left( 0.05\right)$ and a standard deviation of 0.2.

The participant random effects ($\zeta$) were assumed to be drawn from a zero-centred multivariate Normal distribution with 13 dimensions ($\zeta _0^\lambda$, $\zeta _0^\sigma , \hdots , \zeta _5^\sigma$, $\zeta _0^\mu , \hdots , \zeta _5^\mu$), which allows for correlations among the participant random effects. The participant random effects covariance matrix was constructed via a set of half-Normal distributions for the standard deviations and an LKJ distribution (Lewandowski, Kurowicka, & Joe, 2009) for the correlations. The standard deviation for the half-Normal priors was 0.5 for the lapse rate random effect, $\log \left( 2\right) / 2$ for each of the spread parameter random effects, and 1 for each of the midpoint parameter random effects except the intercept (2.5). The shape parameter of the LKJ distribution ($\eta$) was given a value of 2, which applies a weak prior against strong absolute correlations.

The model was implemented in PyMC (version 4.2.2; Salvatier et al. 2016), and Markov chain Monte-Carlo (MCMC) sampling was performed using its implementation of a No-U-turn sampler (Hoffman and Gelman, 2014). A total of 4, 000 draws were used for each of 4 independent chains in the sampling process, after discarding the initial draws (1, 000) used in initializing the sampler.

### Model evaluation

To check how well our statistical model approximated the data, we visually compared the aggregated observed data and the posterior retrodictive samples. The posterior retrodictive samples are generated by drawing samples from the model parameters’ posterior distributions and these samples are then used to generate the simulated data sets. These are also known as posterior *predictive* samples, however we use the term *retrodictive* as the same observed data is used to fit the model (Betancourt, 2020). As can be seen in Fig. 15, the statistical model reproduced the patterns in the observed data with no major discrepancies.

## Appendix B: Experiment 2

### Statistical model

We used a similar statistical modelling strategy to that taken for Experiment 1 in the analysis of Experiment 2. We again assume that participant responses on the main task trials (*y*), whether the average illumination in the scene was judged as being from the right (1) or left (0), can be considered as draws from a Bernoulli distribution:

8 $$\begin{aligned} y_{ijr} \sim \text {Bernoulli}\left( p_{ijr}\right) \end{aligned}$$

where *i* indicates participants, *j* indicates set size conditions, and *r* indicates repeats. We also assume that such responses reflect a mixture of on-task and off-task (lapse) components:

9 $$\begin{aligned} p_{ijr} = \left( 1 - \lambda _i\right) p^t_{ijr} + \lambda _i p^c. \end{aligned}$$

Here, $p^t$ is the probability of a rightwards response based on task-based factors and $p^c$ is the probability of a rightwards response under a lapse (fixed at 0.5). We modelled the number of incorrect responses on the catch trials ($y^c$) as per Experiment 1.

We modelled the probability of a rightwards response based on task-relevant factors ($p^t$) via a ‘wrapped’ cumulative Normal psychometric function ($\Phi _\circ$):

10 $$\begin{aligned} p_{ijr} = \Phi _\circ \left( \theta _{ijr}, \mu _{ij}, \sigma _{ij} \right) . \end{aligned}$$

Here, $\theta$ is the mean of the distribution used to draw the illumination angles for the objects in the stimulus of a given trial, $\mu$ is the midpoint of the function, and $\sigma$ is the standard deviation (spread) of the function. We use the procedure described by Dakin, Mareschal, and Bex (2005a, see their Appendix B) to implement the cumulative distribution function for a wrapped Normal distribution.

We modelled the spread parameter ($\sigma$) via an equivalent noise framework:

11 $$\begin{aligned} \sigma _{ijr} = \sqrt{\frac{\sigma _{\text {int}\left[ ij\right] }^2 + \sigma _{\text {ext}\left[ ijr\right] }^2}{N_j^{b_{ij}}}}. \end{aligned}$$

Here, $\sigma _{\text {ext}}$ and *N* are known values and indicate the amount of external noise on a given trial ($\sigma _{\text {ext}}$) and the number of visible objects on a given trial (*N*), respectively.

We are primarily interested in the number of effective samples used in making the illumination direction judgements, which corresponds to the denominator in the above equation. To assist in appropriately constraining the permissible number of effective samples for a given set size (to between 1 and *N*), we modelled the exponent of the set size (*b*); in this approach, *b* can vary between 0 (corresponding to 1 effective sample) and 1 (corresponding to *N* effective samples), independent of set size. Because *b* is thus constrained to the unit interval, we used a logit link function when modelling *b* via a generalised linear mixed model:

12 $$\begin{aligned} \text {logit}\; b_{ij} = \left( \beta _0^b + \zeta _{0\left[ i\right] }^b\right) + \left( \beta _1^b + \zeta _{1\left[ i\right] }^b\right) x_j. \end{aligned}$$

Here, *x* represents the predictor for the difference in the exponent between the set size conditions; *x* has a value of $-0.5$ for trials with a set size of 9 and a value of $+0.5$ for trials with a set size of 25. The $\beta ^b$ coefficients represent the average exponent across set sizes ($\beta _0^b$) and the difference in exponent between set sizes ($\beta _1^b$), both on a logit scale. The $\zeta ^b$ coefficients are random effects that permits variation across participants.

**Table 2:** Coefficients included in the model for the psychometric function threshold (Eq. 17)

	Incongruent illumination			Congruent illumination		
	Set size: 1	Set size: 9	Set size: 25	Set size: 1	Set size: 9	Set size: 25
$x_1$	+0.5	+0.5	+0.5	-0.5	-0.5	-0.5
$x_2$	-0.5	0.0	+0.5	-0.5	0.0	+0.5
$x_3$	+0.5	-1.0	+0.5	+0.5	-1.0	+0.5
$x_4$	-0.25	0.0	+0.25	+0.25	0.0	-0.25
$x_5$	+0.25	-0.5	+0.25	-0.25	+0.5	-0.25

The amount of internal noise ($\sigma _{\text {int}}$) was modelled using a similar generalised linear mixed model to that of the exponent (*b*), with the exception of the use of a log rather than a logit link function. The midpoint of the psychometric function ($\mu$) was also modelled analogously, using a linear link function.

### Statistical approach

We used a similar statistical approach to that taken for Experiment 1 in the analysis of Experiment 2. We applied a prior of a Student’s *t*-distribution with a mean of 0, a standard deviation of 1.5, and a degrees of freedom of 6 to the average exponent across set sizes parameter ($\beta _0^b$). This use of the degrees of freedom parameter allowed for an increase in the density at the tails of the distribution, which provided a roughly uniform distribution of exponents on the inverse logit (unit interval) scale. The prior on the average amount of internal noise ($\beta _0^{\sigma _{\text {int}}}$) was given by a Normal distribution with a mean of $\log \left( 10\right)$ and a standard deviation of $\log \left( 2\right) / 2$. The specification of the priors for the fixed effects for the lapse rate ($\lambda$) and the midpoint of the psychometric function ($\mu$) were as per Experiment 1.

The participant random effects ($\zeta$) were assumed to be drawn from a zero-centred multivariate Normal distribution with 7 dimensions ($\zeta _0^\lambda$, $\zeta _0^{\sigma _{\text {int}}}$, $\zeta _1^{\sigma _{\text {int}}}$, $\zeta _0^b$, $\zeta _1^b$, $\zeta _0^\mu$, $\zeta _1^\mu$), constructed as per Experiment 1. The standard deviation for the half-Normal priors was 0.5 for each of the exponent ($\zeta ^b$) random effects and $\log \left( 2\right) / 2$ for each of the internal noise ($\zeta ^\sigma _{\text {int}}$) random effects; the priors for the lapse rate ($\zeta ^\lambda$) and midpoint ($\zeta ^\mu$) random effects were as per Experiment 1. The shape parameter of the LKJ distribution ($\eta$) was again given a value of 2.

### Model evaluation

As with Experiment 1, we visually compared the aggregated observed data and the posterior retrodictive samples to check how well our statistical model approximated the data. The statistical model reproduced the patterns in the observed data with no major discrepancies, as can be seen in Figs. 16 and 17.

## Appendix C: Experiment 3

### Statistical model

We used a similar statistical modelling strategy to that taken for Experiments 1 and 2 in the analysis of Experiment 3. We again assume that participant responses on the main task trials (*y*), here whether the shape of the match object was correctly (1) or incorrectly (0) judged as being the same as the shape of the sample object, can be considered as draws from a Bernoulli distribution:

13 $$\begin{aligned} y_{ijkr} \sim \text {Bernoulli}\left( p_{ijkr}\right) \end{aligned}$$

where *i* indicates participants, *j* indicates set size conditions, *k* indicates illumination incongruence conditions, and *r* indicates repeats. We also assume that such responses reflect a mixture of on-task and off-task (lapse) components:

14 $$\begin{aligned} p_{ijkr} = \left( 1 - \lambda _i\right) p^t_{ijkr} + \lambda _i p^c. \end{aligned}$$

Here, $p^t$ is the probability of a correct response based on task-based factors and $p^c$ is the probability of a correct response under a lapse (fixed at 0.5). We modelled the number of incorrect responses on the catch trials ($y^c$) as per Experiments 1 and 2, except that the number of catch trials was 24 per participant in this experiment.

We modelled the probability of a correct response based on task-relevant factors ($p^t$) via a Weibull cumulative distribution function ($\Psi$) that is shifted and scaled such that the probability of a correct response asymptotes at 0.5 as the foil sinusoid amplitude ($\Delta$) approaches zero:

15 $$\begin{aligned} p^t_{ijkr} = 0.5 + 0.5 \times \Psi \left( \Delta _{ijkr}, \nu _{ijk}, \kappa _{i}\right) . \end{aligned}$$

The Weibull cumulative distribution function ($\Psi$) is defined as:

16 $$\begin{aligned} \Psi \left( \Delta , \nu , \kappa \right) = 1 - e^{{-\left( \Delta / \nu \right) }^\kappa }, \end{aligned}$$

where $\nu$ is the threshold and $\kappa$ is the slope of the function.

We are primarily interested in the threshold parameter ($\nu$), which we modelled using a similar approach to the psychometric function parameters in Experiment 1:

17 $$\begin{aligned} \log \nu _{ijk}= & {} \left( \beta _0^\nu + \zeta _{0;i}^\nu \right) x_0\; + \left( \beta _1^\nu + \zeta _{1;i}^\nu \right) x_{1;j} \nonumber \\{} & {} +\left( \beta _2^\nu + \zeta _{2;i}^\nu \right) x_{2;k} + \left( \beta _3^\nu + \zeta _{3;i}^\nu \right) x_{3;k}\nonumber \\{} & {} +\left( \beta _4^\nu + \zeta _{4;i}^\nu \right) x_{4;jk} + \left( \beta _5^\nu + \zeta _{5;i}^\nu \right) x_{5;jk}. \end{aligned}$$

Here, the *x* terms represent the predictors for the intercept ($x_0 = 1$), the main effect of illumination incongruence ($x_1$), the linear ($x_2$) and quadratic ($x_3$) effects of set size rank, and the interaction between illumination incongruence and the linear ($x_4$) and quadratic ($x_5$) effects of set size rank (see Table 2 for the specific predictor values). The $\beta ^\nu$ coefficients represent the fixed effect of each predictor and the $\zeta ^\nu$ coefficients are random effects that permits variation across participants. Note that because we specified $\nu$ in logarithmic units to prevent negative threshold values, the additive effects in Eq. 17 are multiplicative on a linear scale.

We modelled the slope of the psychometric function ($\kappa$) for each participant non-hierarchically and without any main or interaction effects, as our data were not particularly informative of the slope because of its marginalisation in the adaptive staircase procedure. The slope was modelled as:

18 $$\begin{aligned} \log \kappa _i \sim \mathcal {N}\left( \log 10, \log \left( 2\right) / 2\right) \end{aligned}$$

where *i* indexes the participant. The slope was specified in logarithmic units to restrict estimates to positive-only values as we expected the proportion of correct responses to increase as the amplitude of the foil sinusoidal distortion increased.

### Statistical approach

We used a similar statistical approach to that taken for Experiments 1 and 2 in the analysis of this experiment. The prior for the threshold intercept ($\beta _0^\nu$) was a Normal distribution was a mean of $\log (0.075)$ and standard deviation of $\log \left( 2\right) /2$ as we expected the threshold to be around 0.075 across all conditions, with thresholds less than 0.0375 and greater than 0.15 considered unlikely. The prior for the main effect of illumination incongruence ($\beta _1^\nu$) was a skewed-Normal distribution with a location of 0.1, scale of $\log \left( 2\right) /2$, and skew of 3.0, reflecting our belief that varying the direction of illumination between the sample and the match is more likely to worsen than improve performance. The priors for the linear ($\beta _2^\nu$) and quadratic ($\beta _3^\nu$) main effects of set size were each a Normal distribution centered around zero with a standard deviation of $\log \left( 1.5\right) /2$. The priors for the interaction effects ($\beta _4^\nu$ and $\beta _5^\nu$) were each a Normal distribution centered around zero with a standard deviation of $\log \left( 1.25\right) /2$. The priors for the participant random effects on the threshold were each a half-Normal distribution with a standard deviation of $\log \left( 1.5\right) /2$. The priors for the lapse rate ($\zeta ^\lambda$) random effect was as per Experiments 1 and 2. The shape parameter of the LKJ distribution ($\eta$) was again given a value of 2.

### Model evaluation

Consistent with the two previous experiments, we compared the aggregated observed data and the posterior retrodictive samples to check how well our statistical model approximated the data. The statistical model reproduced the patterns in the observed data with no major discrepancies, as can be seen in Fig. 18.

## Open Practices Statement

The study was not preregistered. The raw data, the code used to run the experiments and perform the analyses, and a computational platform (Clyburne-Sherin, Fei, & Green, 2019) for each of the three experiments reported here are available at https://doi.org/10.24433/CO.2101136.v1
